# Developmental RNA-Seq transcriptomics of haploid germ cells and spermatozoa uncovers novel pathways associated with teleost spermiogenesis

**DOI:** 10.1038/s41598-022-18422-2

**Published:** 2022-08-19

**Authors:** Júlia Castro-Arnau, François Chauvigné, Jessica Gómez-Garrido, Anna Esteve-Codina, Marc Dabad, Tyler Alioto, Roderick Nigel Finn, Joan Cerdà

**Affiliations:** 1 Institute of Agrifood Research and Technology (IRTA)-Institute of Biotechnology and Biomedicine (IBB), Universitat Autònoma de Barcelona, 08193 Barcelona, Spain; 2 Institute of Marine Sciences, Spanish National Research Council (CSIC), 08003 Barcelona, Spain; 3 CNAG-CRG, Centre for Genomic Regulation (CRG), Barcelona Institute of Science and Technology (BIST), 08028 Barcelona, Spain; 4 Universitat Pompeu Fabra, 08003 Barcelona, Spain; 5 Department of Biological Sciences, Bergen High Technology Centre, University of Bergen, 5020 Bergen, Norway

**Keywords:** Reproductive biology, Transcriptomics

## Abstract

In non-mammalian vertebrates, the molecular mechanisms involved in the transformation of haploid germ cells (HGCs) into spermatozoa (spermiogenesis) are largely unknown. Here, we investigated this process in the marine teleost gilthead seabream (*Sparus aurata*) through the examination of the changes in the transcriptome between cell-sorted HGCs and ejaculated sperm (SPZ_EJ_). Samples were collected under strict quality controls employing immunofluorescence microscopy as well as by determining the sperm motion kinematic parameters by computer-assisted sperm analysis. Deep sequencing by RNA-seq identified a total of 7286 differentially expressed genes (DEGs) (*p*-value < 0.01) between both cell types, of which nearly half were upregulated in SPZ_EJ_ compared to HCGs. In addition, approximately 9000 long non-coding RNAs (lncRNAs) were found, of which 56% were accumulated or emerged de novo in SPZ_EJ_. The upregulated transcripts are involved in transcriptional and translational regulation, chromatin and cytoskeleton organization, metabolic processes such as glycolysis and oxidative phosphorylation, and also include a number of ion and water channels, exchangers, transporters and receptors. Pathway analysis conducted on DEGs identified 37 different signaling pathways enriched in SPZ_EJ_, including 13 receptor pathways, from which the most predominant correspond to the chemokine and cytokine, gonadotropin-releasing hormone receptor and platelet derived growth factor signaling pathways. Our data provide new insight into the mRNA and lncRNA cargos of teleost spermatozoa and uncover the possible involvement of novel endocrine mechanisms during the differentiation and maturation of spermatozoa.

## Introduction

The developmental remodeling of haploid germ cells (HGCs) into highly polarized spermatozoa capable of motility activation and fertilization is an evolutionary conserved mechanism of male gamete formation^1^. In both mammals and fishes, this spermiogenic differentiation process typically results in the elimination of cytoplasm and organelles together with the morphogenesis of three major compartments, the head containing a condensed haploid nucleus, a mid-section containing the proximal centriole together with one or more mitochondria, and the terminal region composed of an elongated flagellum or flagella^2,3^. Although a variety of specializations evolved in the different lineages, such as the acystic and cystic cellular environments in which mammalian and piscine spermatogenesis respectively progresses, and the absence of an acrosome in most teleost spermatozoa, the overall spermatazoon bauplan is conserved. Similarly, in both lineages, the major endocrine mediators of spermatogenesis are the pituitary follicle-stimulating (FSH) and luteinizing (LH) gonadotropins, that are induced by hypothalamic gonadotropin releasing hormones (GnRH)^4–6^. The LH regulates spermiogenesis indirectly through its cognate LH/choriogonadotropin receptor (LHCGR) to stimulate androgen secretion in the somatic Leydig cells, or also directly through the activation of the LHCGR in the spermatids of teleost fishes^2,4,7^. An understanding of which signaling pathways are activated in the developing germ cells can reveal the conserved or divergent nature of molecular mechanisms regulating vertebrate spermiogenesis and further uncover novel traits associated with spermatozoon formation and fertility.

In mammals, such as boars, bulls, stallions, rodents and primates, transcriptomic studies of spermatozoa or sperm have identified populations of RNAs associated with fertility or that are important for subsequent embryonic development^8–14^. Intricate single-cell RNA-seq studies of cell-sorted germ cells have also identified specific markers of cell populations with significantly distinct transcriptomes, further revealing the conserved and unique spermatogenic features of mice and human^15–17^. By contrast, spermatogenic transcriptomic studies in fishes have been conducted on the whole testes, and therefore these studies have not differentiated between the somatic and germ cell RNA populations^18–33^. As a result, although these works have identified important genes and pathways for spermatogenesis, the genetic network of spermiogenesis remains unexplored.

To gain insight into the molecular regulation of spermiogenesis in fish, the main objective of the present work was to provide a deep characterization of the transcriptome of haploid germ cells (HGC) and ejaculated spermatozoa (SPZ_EJ_) from a modern marine teleost, the gilthead seabream (*Sparus aurata*), by RNA-seq technologies. To achieve this, we employed cell-sorted HGC and strict quality controls of the SPZ_EJ_. By means of this design, our study offers a reliable set of sperm next-generation sequencing data that enable a deeper understanding of the RNA cargo of fish spermatozoa. In addition, in silico analyses of the data uncovered several novel endocrine signaling pathways that may play important roles during the differentiation and maturation of spermatozoa.

## Results

### Isolation and characterization of HGC and SPZ_EJ_

**Figure 1:**
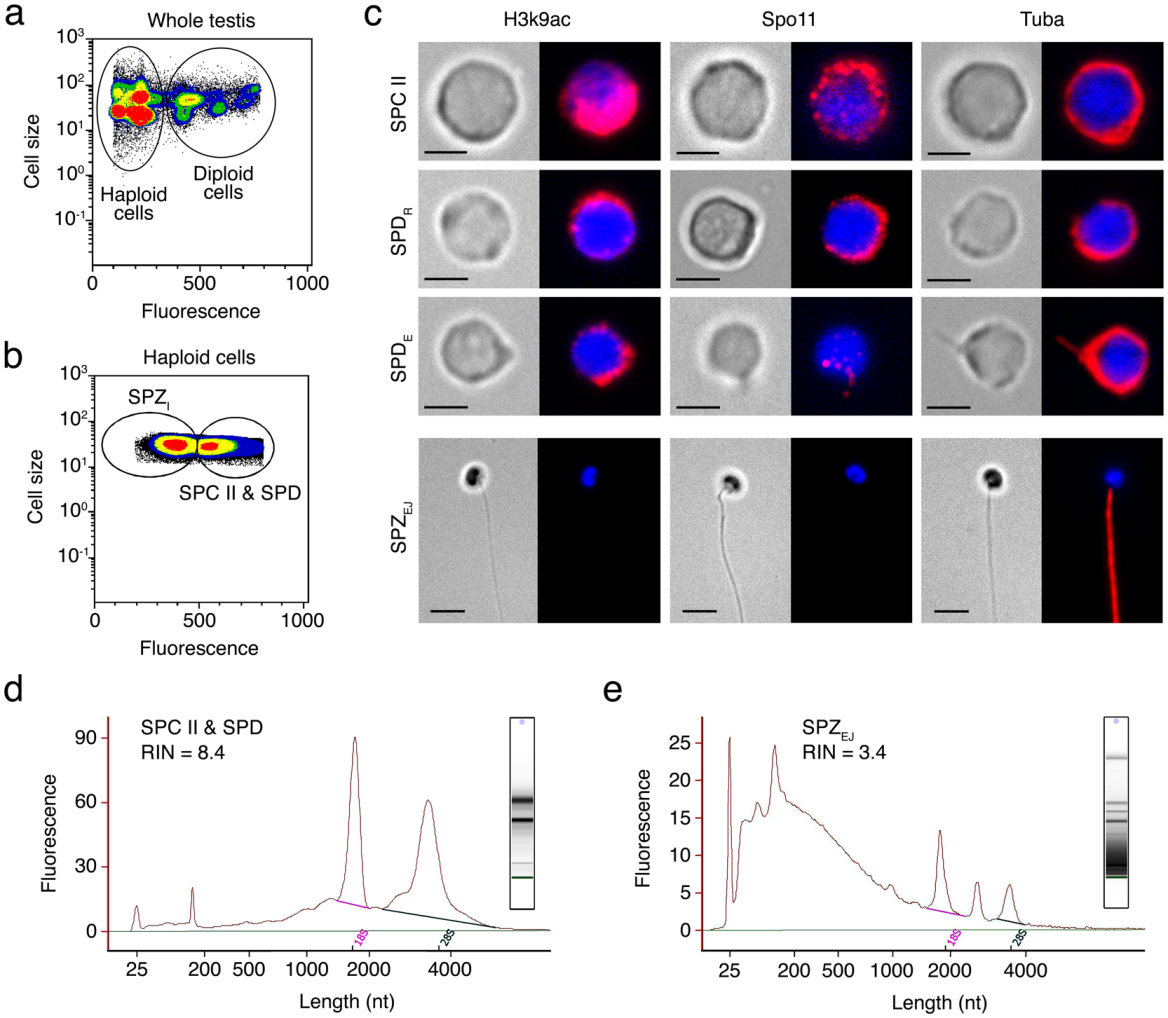
Isolation of testicular haploid germ cells and ejaculated spermatozoa from the seabream and RNA extraction. (**a**, **b**) Representative flow cytometry plots of the seabream cell populations in the whole testis before and after centrifugation generated with the MoFlo XDP cell sorter software (https://www.beckman.es/). In a, the populations of testicular haploid and diploid cells from the testis crude extract before centrifugation are encircled. In b, after centrifugation two subpopulations of haploid germ cells (HGCs) are recovered, one corresponding to a mix of type II spermatocytes (SPC II) and spermatids (SPD), and the other to intratesticular spermatozoa (SPZ_I_). (**c**) Representative immunostaining of Lys^9^ acetylated histone 3 (H3K9ac), meiotic recombination protein Spo11 and α-tubulin (Tuba) in sorted HGC shown in b and ejaculated spermatozoa (SPZ_EJ_). For each cell type the brightfield (left panels) and epifluorescence (right panels) images are shown. SPD_R_, round spermatids; SPD_E_, elongating spermatids. Scale bars, 2 and 5 µm. (**d**, **e**) Electrophoretic size distribution of RNAs in representative samples of SPCII + SPD (**d**) and SPZ_EJ_ (**e**) populations analyzed and plotted by the Agilent Bioanalyzer 2100 Expert Software (https://www.agilent.com/). For each sample, fluorescence is plotted against mRNA length (in nucleotides, nt). The RNA integrity number (RIN) is indicated for each sample.

The changes in gene expression during the differentiation and maturation of seabream spermatozoa were investigated by whole-transcriptome RNA-seq of HGCs and SPZ_EJ_. The HGCs were isolated by fluorescence-activated cell sorting (FACS) from testis samples. Flow cytometry of the extract from the seabream whole mature testis showed the proportion of diploid and haploid cells to be 16% and 84%, respectively (Fig. 1a). The percentage of diploid cells was lower than expected because the centrifugation steps of the extract before cell sorting partially depleted this population. Flow cytometry identified two subpopulations of haploid cells based on their relative size and SYBR Green I fluorescence intensity: a subpopulation formed by spermatocytes II and spermatids (SPC II and SPD, respectively), which we refer here as HGCs, and another subpopulation formed by intratesticular spermatozoa (SPZ_I_) (Fig. 1a, b). The percentage of HGCs and SPZ_I_ in the testicular extracts was of 34 ± 4% and 66 ± 4% (*n* = 15), respectively (Fig. 1b).

Microscopic examination of the HGC-enriched population after FACS confirmed the presence of SPC II, and round and elongating SPD in this fraction (Fig. 1c). Whole-mount immunostaining revealed strong expression of Lys^9^ acetylated histone 3 (H3K9ac) and meiotic recombination protein Spo11 in SPC II, which progressively decreased in round and elongating SPD, and completely vanished in SPZ_EJ_ (Fig. 1c). Immunolocalization of α-tubulin (Tuba) showed that this protein was spread in the cytoplasm in SPC II and round SPD, became detectable in the nascent flagellar region of elongating SPD, and was finally distributed along the flagellum of differentiated SPZ_EJ_ (Fig. 1c). These observations indicate a high occurrence of meiotic recombination in SPC II and a progressive DNA condensation during the differentiation of SPC II into SPD and spermatozoa, which are conserved features during vertebrate germ cell development^34,35^. These data therefore confirmed that the sorted population of cells from the mature seabream testis correspond to HGCs before differentiation into spermatozoa.

The SPZ_EJ_ were collected by manual stripping of naturally spermiating males. The kinematic parameters of the spermatozoa were determined by computer-assisted sperm analysis (CASA) at ~ 2 h after time of collection to assure that the sperm employed for RNA-seq analysis was of high quality. The SPZ_EJ_ selected for further RNA isolation showed a percentage of motility and progressivity at 5 s postactivation from 88 to 98%, and from 16 to 51%, respectively. The time during which spermatozoa remained motile was also monitored and this ranged from 5 to 8 min.

To evaluate the purity and RNA size distribution profiles of the RNA extracted from the HCG and SPZ_EJ_ samples for further RNA-seq analysis, we used the Agilent 2100 Bioanalyzer. The electrophoretic size distribution of extracted RNAs showed that HGC exhibited defined peaks for 18S and 28S ribosomal subunits and high RNA integrity number (RIN) (Fig. 1d, inset). In contrast, in the SPZ_EJ_ extracts the 18S and 28S rRNAs appeared strongly reduced, thus showing a low RIN, which is a typical feature of spermatozoa, together with more abundant short-length mRNA species (Fig. 1e, inset). These data, together with immunofluorescence microscopy examination, indicate that these samples were almost devoid of non-sperm cells^36^.

### Transcriptome profiling of HGC and SPZ_EJ_

Four unstranded RNA libraries (biological replicates) for low-input RNA were subsequently constructed for each of the two HGC and SPZ_EJ_ cell types. The total RNA from HGC and SZP_EJ_ was extracted from 12 males, and each of the four libraries was constructed from a pool of three different males. Library sequencing rendered 30–62 million reads per library corresponding to a yield of 5–10 Gb per library. From these data, we produced a new integrative *S. aurata* genome annotation before the RNA-seq analysis. This new annotation was carried out by re-annotating the available *S. aurata* reference genome^37^, and by adding 202 de novo assembled transcripts that were not present in the genome assembly. In total, 31,501 protein-coding genes were annotated, which produced 57,396 transcripts (1.82 transcripts per gene) that encode for 51,365 unique protein products. Functional labels were assigned to 62% of the annotated proteins. In addition, 165,898 non-coding transcripts were annotated, of which 159,925 are long non-coding RNAs (lncRNAs) and 5973 correspond to short non-coding RNAs.

**Figure 2:**
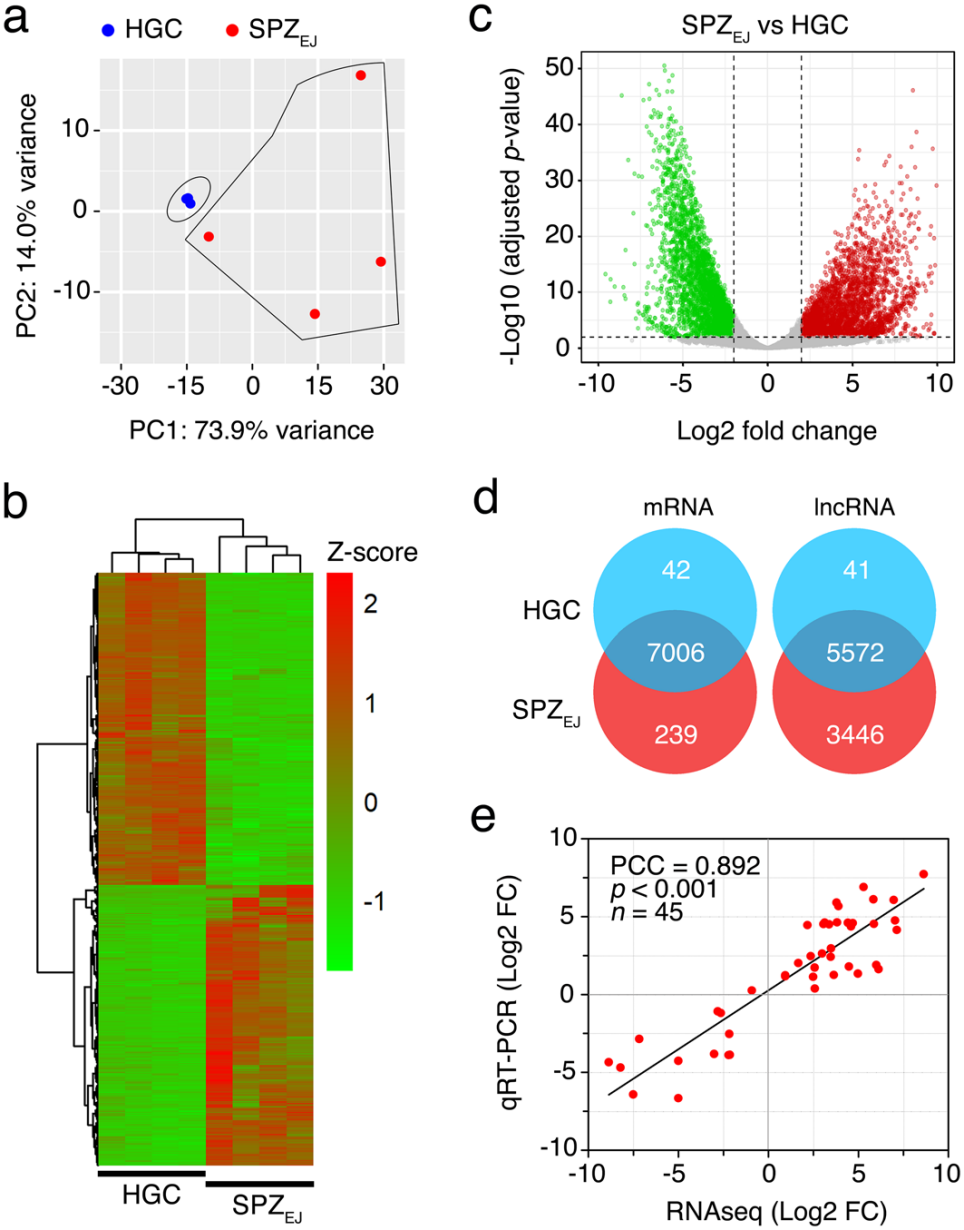
RNA-seq data analysis of seabream HGC and SPZ_EJ_. (**a**) Principal component analysis (PCA) using the top 500 most variable genes between HGC and SPZ_EJ_ (*n* = 4 pools) and the 'rlog' transformation of the counts. (**b**) Heatmap generated by unsupervised hierarchical clustering of RNAseq expression z-scores computed for the 7286 differentially expressed genes (DEGs) (*p*-adj < 0.01; Log2 fold change > 1) between HGC and SPZ_EJ_. The heatmap was generated with the ‘pheatmap’ R package (https://CRAN.R-project.org/package=pheatmap). (**c**) Volcano plot representation of DEGs in the SPZ_EJ_ versus HGC comparison. The x-axis shows Log2 fold changes in expression and the y-axis the negative logarithm of their *p*-value to base 10. Red and green points mark the genes with significantly increased or decreased expression respectively in SPZ_EJ_ compared to HGC (FDR < 0.01). (**d**) Venn diagrams showing the number of HGCs- and SPZ_EJ_-specific and common mRNAs and lncRNAs (in intersect region) which are differentially expressed between the two stages. The Venn diagrams and volcano plots were performed with the ‘VenDiagramm’ R package (https://CRAN.R-project.org/package=VennDiagram) and ‘ggplot2’ R package (https://ggplot2.tidyverse.org), respectively. (**e**) Validation of the RNAseq data by qRT-PCR. The plot represents the Pearson's correlation analysis of DEGs in HGC and SPZ_EJ_ determined by RNAseq and qRT-PCR. The Pearson’s correlation coefficient (PCC) of the Log2 fold change analyzed by RNAseq (x-axis) and using qRT-PCR (y-axis), the *p*-value, and the number of DEGs analyzed are indicated. Plots in a, d and e were generated using the GraphPad Prism v8.4.3 (686) software (https://www.graphpad.com/).

Principal component analysis (PCA) of the expression data showed that FACS-purified HGC and SPZ_EJ_ formed two relatively well-separated clusters, suggesting that the developmental stage has a large effect on the pattern of gene expression (Fig. 2a). However, while the four HGC biological replicates clustered very closely, those of SPZ_EJ_ were more distant, indicating a higher variability in the transcriptome of the SPZ_EJ_ replicates. Nevertheless, the RNA-seq analysis revealed a total of 7286 differentially expressed genes (DEGs) (adjusted *p*-value < 0.01) between both cell types, of which nearly half (3446) were upregulated in SPZ_EJ_ when compared to HGCs (Fig. 2b–d). In addition, 239 transcripts were detected only in SPZ_EJ_ (Fig. 2d), indicating that they emerged de novo in the spermatozoa during the differentiation and maturation phases. The top ten upregulated mRNAs included transcripts potentially involved in spermatogenesis, sperm capacitation, oxidative stress, or sperm-egg interaction, such as *tlr1*, *zp*, *tyro3* and *alox15b*, as well as other genes with potential functions in transcription, cell adhesion, binding and presentation of antigens, or mitochondrial permeability (*mafb*, *apbb1*, *cdc209c*, *fhl2*, *h2-aa* and *arhgap11b*) (Table 1). Finally, we also found a high number of differentially expressed lncRNAs (9059 sequences) of which 5114 were upregulated in SPZ_EJ_ (Fig. 2d), which indicates that 56% of the total identified lncRNAs were accumulated in SPZ_EJ_. In addition, most of the upregulated lncRNAs (67%) were unique in SPZ_EJ_.

**Table 1:** Top 10 upregulated mRNAs in SPZ_EJ_, their fold-change in expression with respect to HGC and mean of fragments per kilo base of transcript per million mapped fragments (FPKM), and their potential associated functions according to GeneCards (www.genecards.org).

Product	Gene	Sequence ID	Log2 FC	Mean FPKM	Reproductive-related function
Toll-like receptor 1	*tlr1*	XM_030396315.1	11.16	1698	Involved in ovulation, sperm capacitation and fertilisation
Zona pellucida protein X, partial	*zp*	AAY21008.1	11.02	657	Sperm-egg interaction
Transcription factor MafB	*mafb*	XP_030276052.1	10.69	558	Acts as a transcriptional activator or repressor that may be involved in spermatogenesis. Unknown function in spermatozoa
Amyloid beta A4 precursor -binding family B member 1	*apbb1*	XP_030254479.1	10.08	236	Transcription coregulator that can have both coactivator and corepressor functions. Can bind modified histones and chromatin modifying enzymes, thereby regulating transcription
CD209 antigen C	*cd209c*	XP_030265713.1	10.05	839	C-type lectin that functions in cell adhesion and pathogen recognition. Unknown function in spermatozoa
Four and a half LIM domain 2	*fhl2*	XP_030299463.1	10.03	226	May function as a molecular transmitter linking various signaling pathways to transcriptional regulation. Unknown function in spermatozoa
H-2 class II histocompatibility antigen, A-Q alpha chain	*h2-aa*	XP_030263565.1	9.96	1644	Binding and presentation of peptides derived from antigens. Unknown function in spermatozoa
Tyrosine-protein kinase receptor TYRO3	*tyro3*	XP_030287857.1	9.88	308	Involved in spermatogenesis. Unknown function in spermatozoa
Arachidonate 15-lipoxygenase B	*alox15b*	XP_030274670.1	9.83	212	Catalyzes the peroxidation of free and esterified polyunsaturated fatty acids (PUFAs) generating a spectrum of bioactive lipid mediators. Involved in the oxidative stress cascade of spermatozoa
Inactive Rho GTPase-activating protein 11B	*arhgap11b*	XP_030261033.1	9.83	647	Inhibits the mitochondrial permeability transition pore (mPTP), thereby increasing mitochondrial Ca^2+^. Unknown function in spermatozoa

The quality of the RNA-seq data and the reliability of the DEGs identified were validated on randomly selected 45 DEGs by real-time quantitative reverse transcription PCR (qRT-PCR) in three biological replicates. Fold changes from qRT-PCR were compared with the RNA-seq expression profiles (Fig. 2e). The dynamic expression patterns of all genes were consistent with the RNA-seq analysis, showing a high correlation (Pearson’s correlation coefficient of 0.892) between RNA-seq and qRT-PCR data. These results therefore indicated the reliability of the RNA-seq for mRNA differential expression analysis.

### Functional enrichment analysis of DEGs during spermatozoa differentiation and maturation

Gene ontology (GO) term-enriched analysis of DEGs in SPZ_EJ_ with significant differences revealed that a large number of biological processes were represented. The five top-ranked GO terms in biological processes were regulation of biological, cellular and metabolic processes, and organic substance and metabolic processes (Suppl. Fig. S1a). Further analysis of GO term distribution indicated that the most represented biological process was the regulation of gene expression, followed by positive regulation of macromolecule and cellular metabolism, regulation of signal transduction, and regulation of cellular biosynthesis (Suppl. Fig. S1b). Interestingly, genes with GO terms such as cellular response to stimulus, cell communication, signal transduction, response to external or chemical stimulus, cell adhesion, and cell surface receptor signaling pathway, were only upregulated in SPZ_EJ_ (Suppl. Fig. S1a and S1b). For the GO molecular function, the top enriched terms were binding to ribonucleotides and purine nucleotides, whereas the terms Ca^2+^, phosphatidylinositol and actin binding, ion channel activity, and transmembrane transport of inorganic cations and organic anions appeared to be only upregulated in SPZ_EJ_ (Suppl. Fig. S1c). Taken together these findings indicate the enrichment of gene expression, metabolic and signaling processes in SPZ_EJ_.

**Figure 3:**
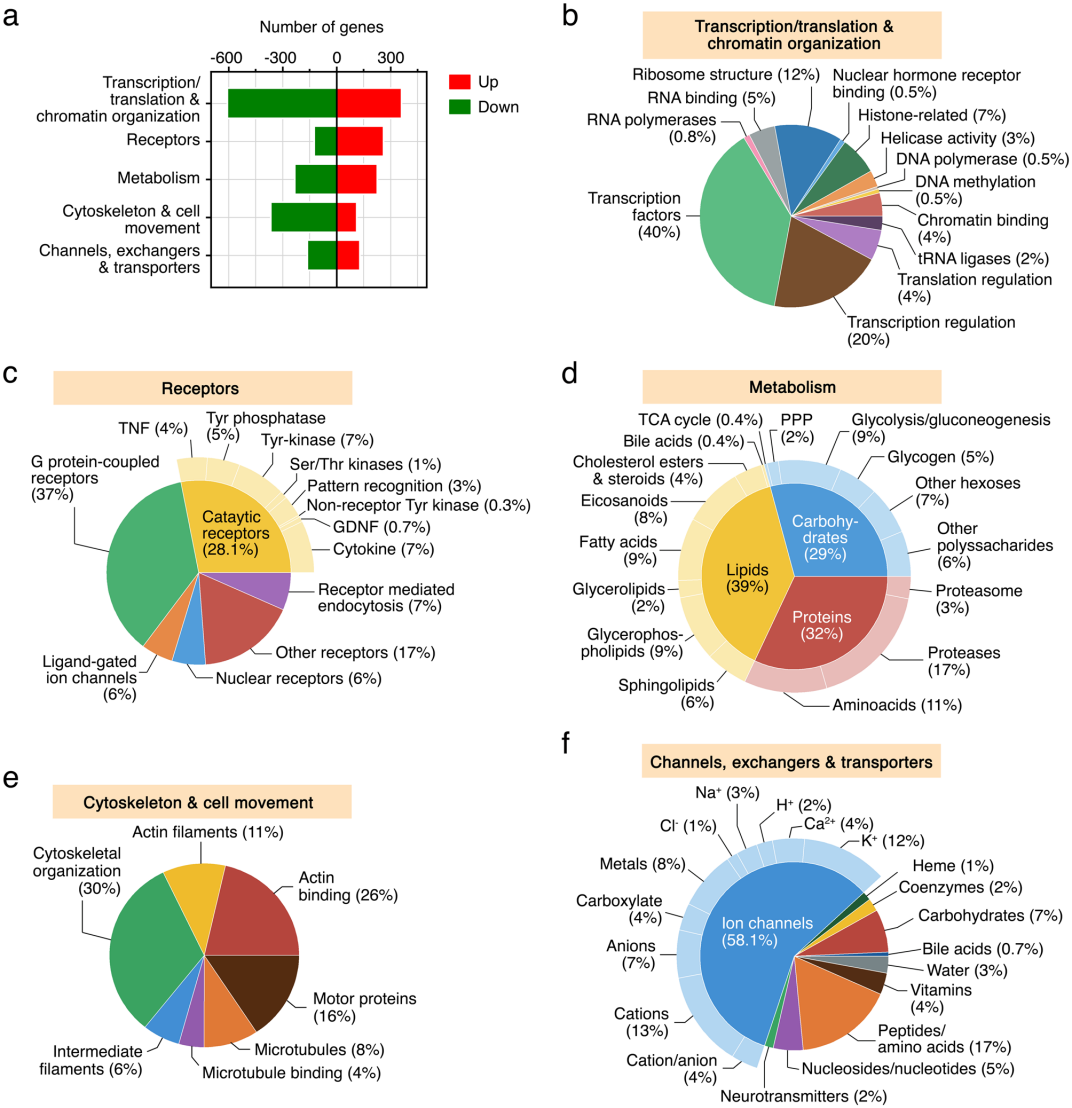
Functional classification of DEGs during sperm differentiation and maturation. (**a**) Transcriptional regulation of a subpopulation of DEGs classified into five functional categories: transcription and translation and chromatin organization, receptors, metabolism, cytoskeleton and cell movement, and channels, exchangers and transporters. (**b**–**f**) Pie charts showing the GO term distribution of upregulated DEGs in SPZ_EJ_ included in each of the five functional groups. The numbers are the percentage of genes in each category. Plots were generated using the GraphPad Prism v8.4.3 (686) software (https://www.graphpad.com/).

To gather more information on genes with a potential impact on spermatozoa function, the DEGs in SPZ_EJ_ were manually classified into five functional categories by using GO analysis and the Uniprot database. These categories included transcription, translation and chromatin organization (978 genes), receptors (395 genes), metabolism of proteins, lipids and carbohydrates (469 genes), cytoskeleton and cell movement (487 genes), and channels, exchangers and transporters (303 genes) (Fig. 3a and Suppl. Table S1). The genes upregulated in SPZ_EJ_ related to transcription, translation and chromatin organization (365 genes) mainly correspond to transcription factors (41%) and regulators of transcription (21%), followed by structural constituents of ribosomes (12%), regulators of translation (4%), chromatin and RNA binding (4 and 5%, respectively), and histones and histone modification (7%) (Fig. 3b). Most of the highest upregulated genes of this group (log2 fold change > 5) correspond to transcriptional regulators, including transcription factors, but other mRNAs encoding late histone H2A.2.2 and H2B.L4-like (*h2a*.2.2 and *h2b.l4*), helicase with zinc finger domain 2 isoform X1 (*helz2*), or DNA (cytosine-5)-methyltransferase 3B (*dnmt3b*), were also highly accumulated in SPZ_EJ_ (Suppl. Table S1). Interestingly, ~ 25% of these highly upregulated genes are possibly involved in activation rather than repression of transcription, whereas ~ 29% of them can potentially repress or activate transcriptional activity.

Most of the receptor-encoding upregulated genes (266 genes) were G protein-coupled receptors (37%), tyrosine phosphatase and kinase receptors (11%), cytokine receptors (7%), as well as other receptors mainly including glycoprotein, Fc, and scavenger receptors (17%) (Fig. 3c). The highest expressed genes in each of these groups were lysophosphatidic acid receptor 6 (*lpar6*), potentially involved in protection of oxidative stress, receptor-type tyrosine- phosphatase C (*ptprc*), interleukin-1 receptor type 1 (*il1r1*), and zona pellucida sperm-binding protein 3 (*zp3*), likely implicated in the sperm-egg interaction (Suppl. Table S2).

For the upregulated genes encoding metabolic components (230 genes), those related to the metabolism of lipids, proteins and carbohydrates were almost equally represented (39, 32 and 29%, respectively) (Fig. 3d). The most enriched genes in SPZ_EJ_ belonging to these groups were those related to glycerophospholipid hydrolysis and fatty acid biosynthesis (18%), such as lysophosphatidylserine lipase ABHD12 (*abhd12*) and elongation of very long chain fatty acids 1 (*elovl1*), proteases (17%), such as transmembrane protease serine 9 (*tmprss9*), and glycolysis and gluconeogenesis (9%), such as triosephosphate isomerase (*tpi1*), which synthesizes D-glyceraldehyde 3-phosphate from glycerone phosphate at the beginning of the glycolytic pathway (Fig. 3d and Suppl. Table S3).

The genes encoding for proteins involved in cytoskeletal organization (30%), actin binding (26%) and molecular motors (16%) were the most abundant upregulated genes involved in the category of cytoskeleton and cell movement (116 genes) (Fig. 3e). The highest upregulated genes, however, correspond to actin binding, such as myristoylated alanine-rich C-kinase substrate (*marcks*), and motor proteins or components of the motor-adapter complex, such as kinesin Kif20a (*kif20a*) and syntabulin (*sybu*) (Suppl. Table S4).

Finally, in the group including genes encoding for channels, exchangers and transporters (134 genes), more than a half of the upregulated genes encode for ion channels (58%), whereas the rest included water channels (3%) and transporters of peptides and amino acids (17%), carbohydrates (7%), nucleosides and nucleotides (5%), vitamins (4%), neurotransmitters (2%) and bile acids (0.7%) (Fig. 3f). The K^+^ and metal specific channels (20%), such as potassium voltage-gated channel subfamily K members 6 and 4 (*kcnk6* and *kcng4*) and transmembrane channel 7 (*tmc7*), as well as cation channels (13%), such as solute carrier family 22 member 5-like (*slc22a5*), were the most enriched in SPZ_EJ_ (Fig. 3f and Suppl. Table S5). Interestingly, the water and glycerol-facilitating channel aquaporin-7 (*aqp7*), which was previously described in seabream spermatozoa^38^, and the solute carrier family 43 member 3 (*slc43a3*), which is a sodium-independent purine-selective nucleobase transporter, were the most upregulated mRNAs in SPZ_EJ_ from the whole group of channels, exchangers and transporters (log2 fold change of 9.58 and 8.85, respectively) (Suppl. Table S5), suggesting an important role of these genes during the differentiation and maturation of spermatozoa.

### Protein–protein interaction analyses

**Figure 4:**
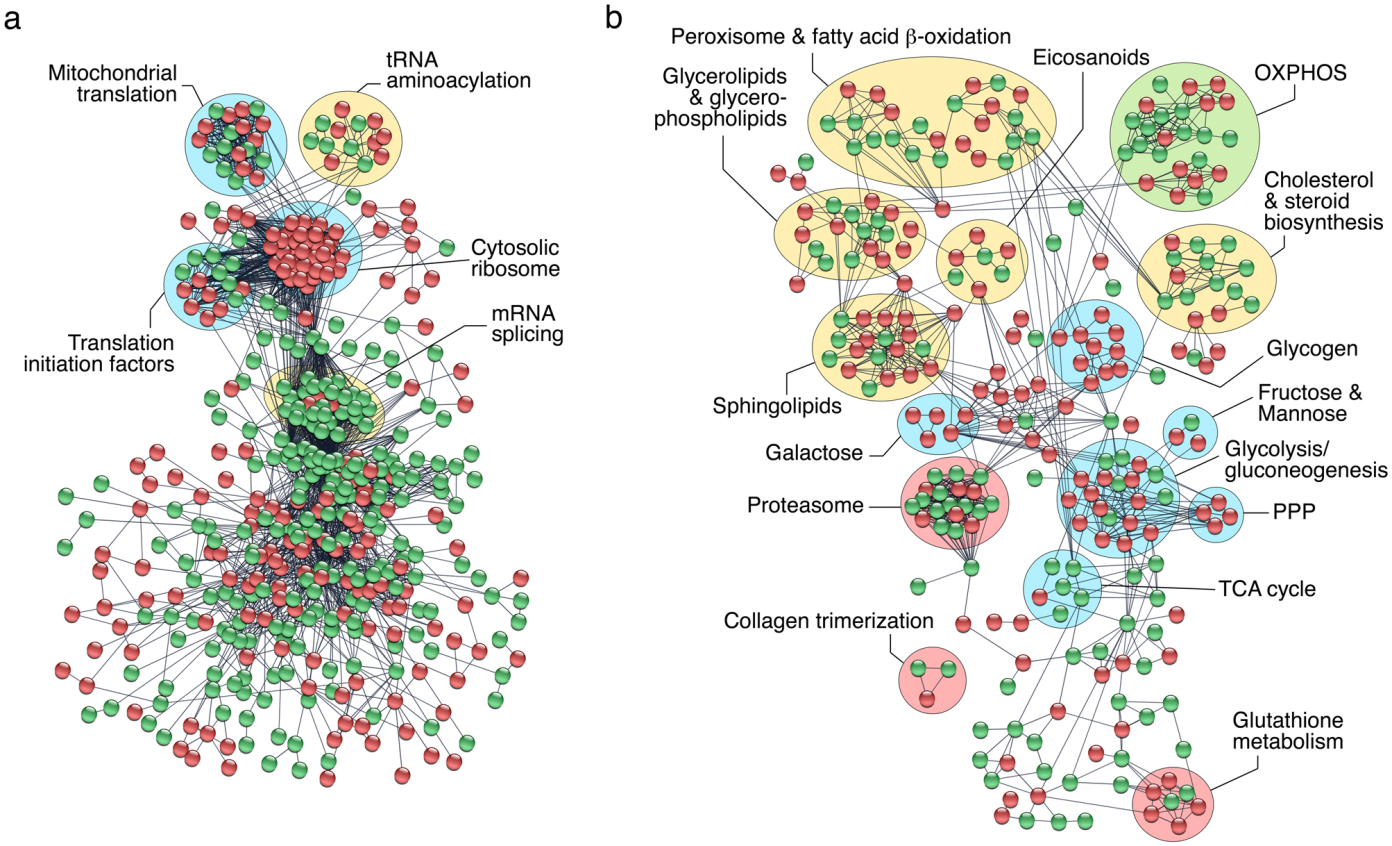
Protein–protein interaction (PPI) networks of DEGs. The PPI information of DEGs potentially involved in transcription and translation and chromatin organization (**a**), and metabolism (**b**), was obtained through a database search using STRING database v11 with a high confidence score (0.9), and imported into Cytoscape v3.8.2 (https://cytoscape.org/) for network construction and plotting. Proteins and their interactions are shown as nodes (spheres) and edges (lines), respectively. Nodes in red or green color indicate upregulated and downregulated DEGs, respectively. Proteins are grouped based on their known biological functions. OXPHOS, oxidative phosphorylation; PPP, pentose phosphate pathway; TCA, tricarboxylic acid.

In an effort to identify specific transcription/translation and carbohydrate metabolic processes enriched in SPZ_EJ_, we built a putative protein interactome network of DEGs classified into these two categories by using the STRING protein–protein interaction (PPI) database for known PPIs^39^ under very stringent inclusion criteria. As a result, a connected network comprising 766 proteins and 3588 connections was mapped for the proteins encoded by genes involved in transcription and translation (Fig. 4a). These proteins could be divided into five major subclusters based on their known biological functions established through GO analysis, including mitochondrial translation, tRNA aminoacylation, translation initiation, cytosolic ribosomes, and mRNA splicing (Fig. 4a). All of the DEGs grouped into the cytosolic ribosome subunit subcluster, and half of the DEGs belonging to the transfer RNA (tRNA) aminoacylation, mitochondrial translation, and translation initiation subclusters, were upregulated (Fig. 4a). These findings, together with the previous observation of the relatively high abundance of transcription activators among the upregulated genes in SPZ_EJ_, suggest that both transcription and mitochondrial and cytoplasmic translation activity may occur during the differentiation and maturation of spermatozoa.

**Figure 5:**
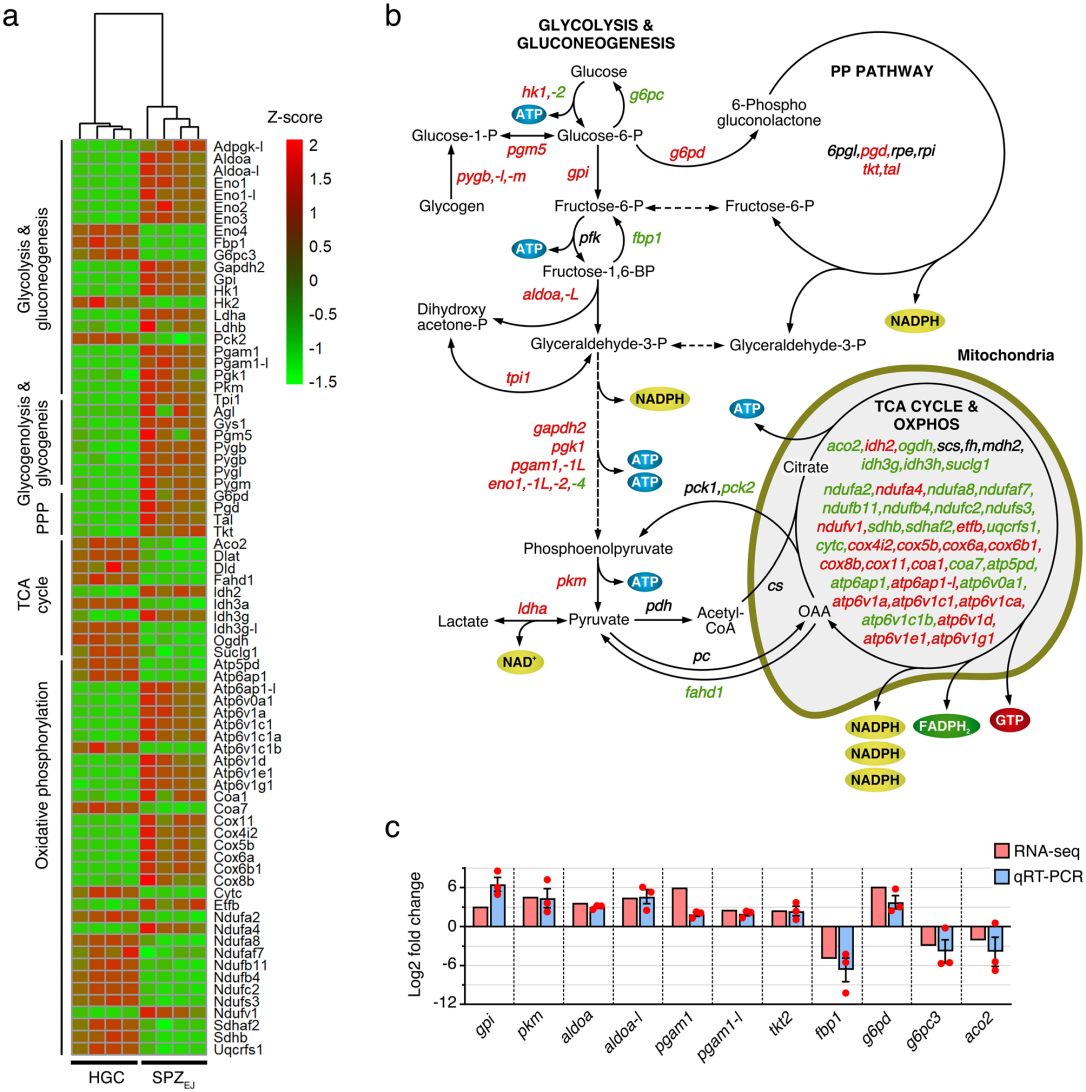
Analysis and mapping of DEGs potentially involved in respiratory pathways in seabream spermatozoa. (**a**) Hierarchical clustering heatmap of RNA-seq expression z-scores computed for genes encoding enzymes potentially involved in carbohydrate metabolism, penthose phosphate pathway (PPP), tricarboxylic acid (TCA) cycle, and oxidative phosphorylation (OXPHOS), that are differentially expressed (*p*-adj < 0.01; Log2 fold change > 1) between HGC and SPZ_EJ_. The map was generated with the ‘pheatmap’ R package (https://CRAN.R-project.org/package=pheatmap). (**b**) Schematic diagram of the biochemical pathways of glycolysis/gluconeogenesis, PPP, TCA cycle and OXPHOS. Enzyme-coding DEGs in green and red color denotes downregulation and upregulation, respectively, whereas black color indicates no change in the expression levels. The diagram was generated using CorelDRAW Graphics Suite 2021.5 (https://www.coreldraw.com/). (**c**) Validation of the changes in expression of 11 selected genes classified into respiratory pathways by qRT-PCR. Data from qRT-PCR are the mean ± SEM (*n* = 3 pools of 3 different fish each) and were plotted using the GraphPad Prism v8.4.3 (686) software (https://www.graphpad.com/).

The metabolism interactome showed 379 proteins and 821 connections divided into fifteen subclusters, from which those corresponding to glycolysis/gluconeogenesis, the pentose phosphate (PP) pathway and sphingolipid, galactose, glycogen and glutathione metabolism, were the most upregulated in SPZ_EJ_ (Fig. 4b). Further mapping of the 76 DEGs coding for enzymes involved in respiratory pathways, as well as qRT-PCR validation of a few of these transcripts, indicated that most of the genes of the tricarboxylic acid (TCA) cycle, as well as three genes coding for specific enzymes of gluconeogenesis, such as phosphoenol-pyruvate carboxykinase (*pck2*), fructose 1,6-bisphosphatase (*fbp1*) and glucose 6-phosphatase (*g6pc*), were downregulated or not differentially expressed in SPZ_EJ_ (Fig. 5a–c). In contrast, half of the genes involved in the PP pathway, such as 6-phosphogluconate dehydrogenase (*pgd*), transketolase (*tkt*) and transaldolase (*tal*), and most of the glycolytic enzyme-encoding genes, including the two key enzymes hexokinase-1 (*hk1*) and pyruvate kinase (*pkm*), were upregulated in SPZ_EJ_ (Fig. 5a–c). Similarly, many of the genes coding for enzymes catalyzing oxidative phosphorylation (OXPHOS) were also upregulated in SPZ_EJ_ (Fig. 5a–c). These data therefore suggest that both glycolysis and OXPHOS are important pathways for ATP generation in seabream spermatozoa.

### The cytokine, PDGF and GnRHR signaling pathways are highly upregulated in SPZ_EJ_

**Figure 6:**
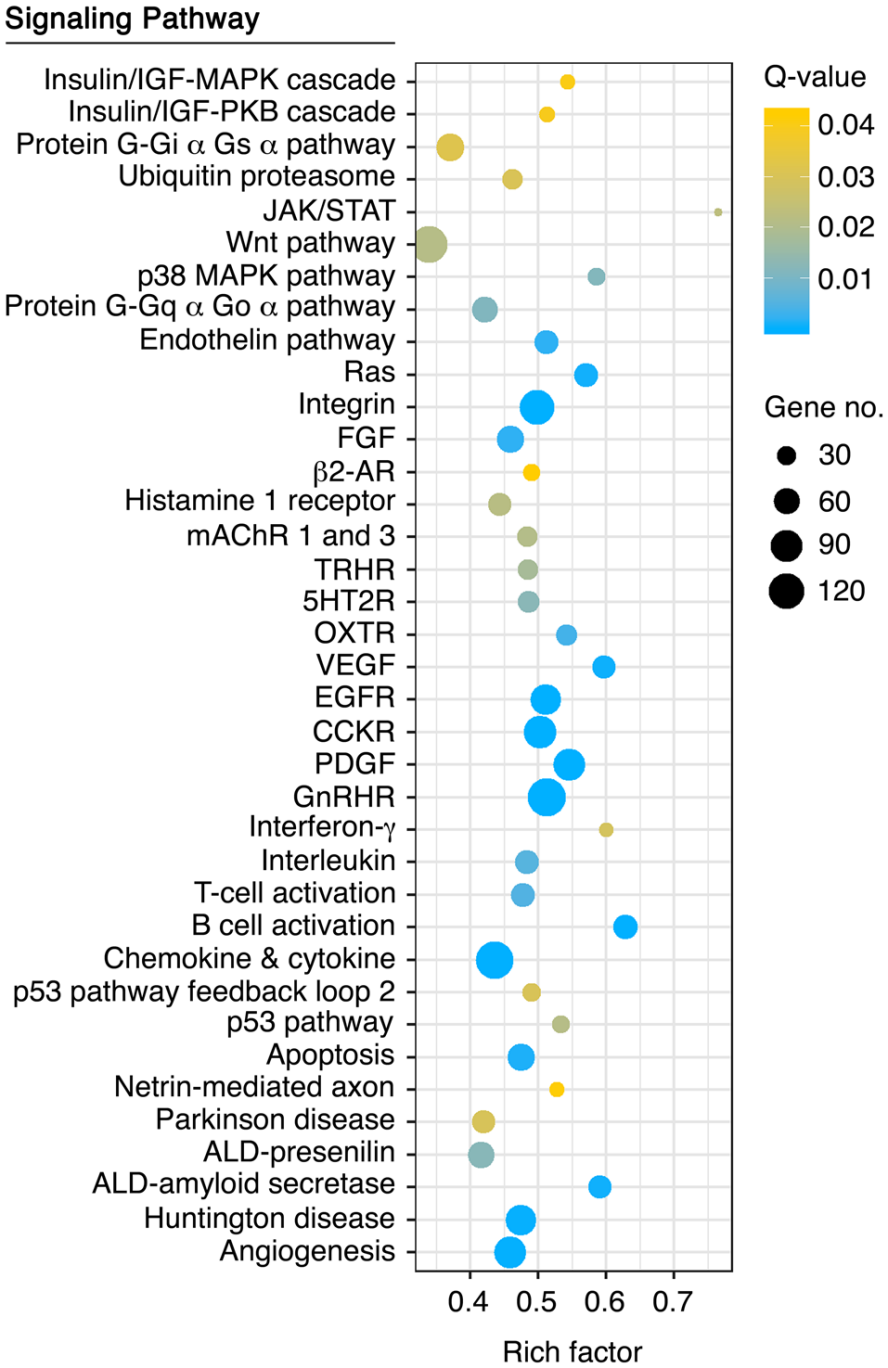
Pathway enrichment analysis among DEGs during spermatozoa differentiation and maturation using the PANTHER Classification System. The plot generated with the ‘ggplot2’ R package (https://ggplot2.tidyverse.org) shows the 37 most highly enriched signaling pathways (FDR < 0.05) in SPZ_EJ_. The left Y-axis shows the PANTHER pathway, whereas the X-axis shows the rich factor. A high Q-value is represented by yellow and a low Q-value is represented by blue.

In order to identify signaling pathways enriched in SPZ_EJ_, pathway analysis was carried out for the 7287 DEGs using the PANTHER classification system^40^. The analysis identified a total of 960 transcripts belonging to 37 different signaling pathways, including 13 receptor pathways (Fig. 6). Highly enriched and significant pathways were integrin, epidermal growth factor receptor (EGFR), fibroblast growth factor (FGF), cholecystokinin receptor (CCKR), rat sarcoma virus (Ras), vascular endothelial growth factor (VEGF), and B cell activation. However, amongst the most dominant pathways regulated in SPZ_EJ_ in terms of number of genes identified, rich factor and lowest *p*-values were the inflammation mediated by chemokine and cytokine, PDGF and GnRHR signaling pathways (Fig. 6). Mapping of these DEGs, as well as of other transcripts detected but not regulated in SPZ_EJ_, on the corresponding KEGG pathway and WikiPathways databases showed that most components of the chemokine and cytokine, PDGF and GnRH signaling pathways were expressed in SPZ_EJ_ (Suppl. Figs. S2–S4).

**Figure 7:**
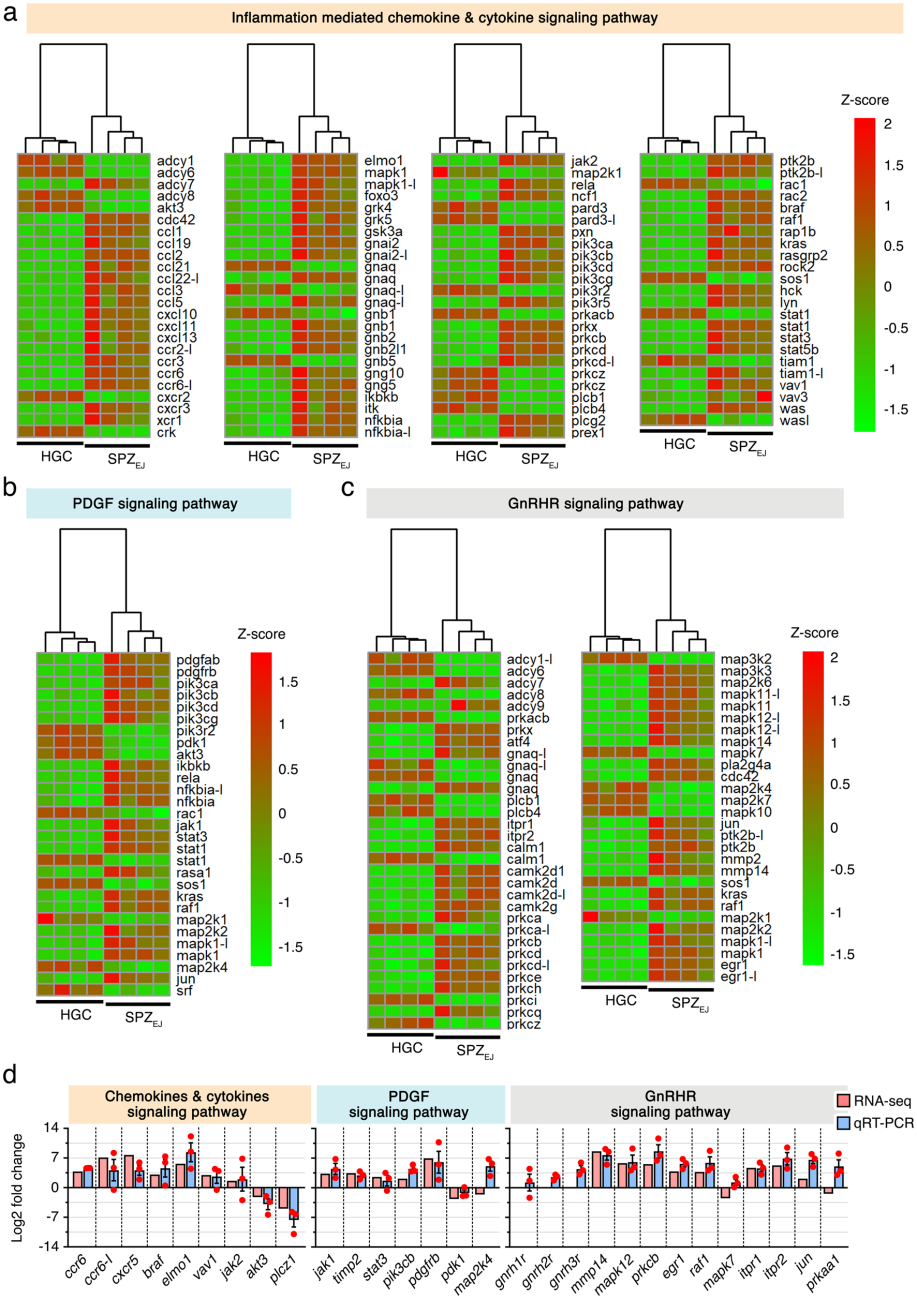
Hierarchical clustering heatmaps of RNA-seq expression for genes potentially involved in the inflammatory chemokine and citokine (**a**), PDGF (**b**) and GnRHR (**c**) signaling pathways. The maps were generated with the ‘pheatmap’ R package (https://CRAN.R-project.org/package=pheatmap). (**d**) qRT-PCR validation of the changes in expression of selected genes classified into these pathways. Data from qRT-PCR are the mean ± SEM (*n* = 3 pools of 3 different fish each) and were plotted using the GraphPad Prism v8.4.3 (686) software (https://www.graphpad.com/).

Hierarchical clustering heatmaps showed that most of the genes related to the chemokine and cytokine, PDGF and GnRHR pathways were upregulated in SPZ_EJ_ (Fig. 7a–c). Thus, for the chemokine and cytokine pathway the most accumulated transcripts (log2 fold change > 7) were C-X-C motif chemokine 10 (*cxcl10*), C-C chemokine receptor type 6 (*ccr6*), C-C motif chemokine 3 and 19 (*ccl3* and *ccl19*), and ras-related C3 botulinum toxin substrate 2 (*rac2*) (Fig. 7a). For the PDGF pathway, the Pdgf receptor b (*pdgfrb*), phosphatidylinositol 4,5-bisphosphate 3-kinase catalytic subunit delta isoform (*pik3cd*), inhibitor of nuclear factor kappa-B kinase subunit beta (*ikbkb*), rapidly accelerated fibrosarcoma (RAF) proto-oncogene serine threonine-protein kinase (*raf1*), and signal transducer and activator of transcription 1 (*stat1*), were the highest upregulated genes (log2 fold change > 4) (Fig. 7b). Finally, in the GnRHR pathway the highest expressed genes (log2 fold change > 6) were matrix metallopeptidase-2 and -14 (*mmp2* and *mmp14*), mitogen-activated kinase 12 (*mapk12*), calcium calmodulin-dependent kinase type II delta 1 chain (*camk2d1*) and adenylate cyclase type 9 (*adcy9*) (Fig. 7c). These data were validated by qRT-PCR for a number of genes from each pathway, including three Gnrhrs identified in our transcriptome (*gnrhr1*, *gnrhr2* and *gnrhr3*) for which the RNA-seq did not detect significantly different expression levels (Fig. 7d). The qRT-PCR analysis showed however that both *gnrhr2* and *gnrhr3* are in fact upregulated in SPZ_EJ_, whereas the *gnrhr1* is not (Fig. 7d). Altogether, these data suggest the activation of the chemokine and cytokine, GnRHR and PDGF signaling pathways during seabream spermiogenesis.

### Transcripts expressed de novo in SPZ_EJ_

We finally investigated the 236 transcripts that were only identified in SPZ_EJ_, which were considered as emerging de novo during the differentiation and maturation of spermatozoa. These transcripts were classified into different functional categories by using GO analysis and the Uniprot database, which showed that those potentially involved in recognition, binding or adhesion processes of cells, cell signaling, metabolism, and transcription and translation, were the most abundant (21%, 17%, 14% and 13%, respectively) (Suppl. Fig. S5a). Interestingly, among these transcripts, the CD209 antigen (*cd209*) (mean FPKM = 231), toll-like receptor 13 (*tlr13*) (mean FPKM = 38), tetraspanin-13 (*tspan13*) (mean FPKM = 21), guanine nucleotide-binding G(I) G(S) G(T) subunit beta-2 (*gnb2*) (men FPKM = 10), tumor necrosis factor alpha-induced protein 8-like protein 3 (*tnfaip8l3*) (mean FPKM = 17), gamma-glutamyltransferase 5-like (*ggt5*) (mean FPKM = 14), and transcription factor jun-D-like (jund) (mean FPKM = 12) showed the highest expression in SPZ_EJ_ (Suppl. Fig. S5a). Further interactome analysis of the proteins potentially encoded by the de novo transcripts revealed the upregulation of components of the Ca^2+^ and cAMP, and phosphatidylinositol 3-kinase (PI3K/Akt), signaling pathways in SPZ_EJ_ (Suppl. Fig. S5b), suggesting an important role of these pathways for spermatozoon function. In addition, gene set enrichment and pathway analyses among the de novo genes in SPZ_EJ_ confirmed the activation of the GnRHR and PDGF signaling pathways, and also suggested a potential role of heterotrimeric G proteins-mediated signal transduction mechanisms in SPZ_EJ_ (Suppl. Fig. S6).

## Discussion

The present study reports for the first time to our knowledge the molecular signature of spermiogenesis in a teleost fish. By combining flow cytometry and cell sorting with transcriptomics, we were able to identify key candidate genes and functional pathways that may be essential to sperm function and early embryo development. Our data also support the view that the teleost sperm transcriptome is a complex and heterogeneous network of coding and non-coding RNA molecules as previously noted in the human sperm^14^.

The role of lncRNAs on mammalian spermatogenesis has been predicted, but functional studies exploring their mechanism of action and relevance to sperm function are lacking^41^. In the human spermatozoon the lncRNA cargo of the is of about 7521 sequences^14^, thus close to the number of lncRNAs found in seabream SPZ_EJ_ in our study (8560). In human, most of the lncRNAs detected in the spermatozoon were categorized as antisense and long intervening/intergenic noncoding RNAs (lincRNAs), which frequently regulate gene expression in *cis*^14,42^. The lncRNAs cargo of human and seabream spermatozoa is possibly understimated since the workflow employed in both studies allowed the inclusion of coding and non-coding transcripts with a selection of poly(A) RNAs, which enriched the RNA-seq libraries in polyadenylated antisense lncRNAs^43^. In mammals, many targets of spermatozoon lncRNAs have been predicted^44^, and found to be enriched in apoptosis (e.g., PI3K-AKT, p53)^45^, capacitation-related pathways (e.g., Ca^2+^, cAMP, and MAPK signaling)^46^, motility^47–49^, and first stages of embryo development^14^. In teleosts, such information is still lacking, but our present findings show that the number of de novo emerging lncRNAs in mature seabream sperm is much higher than that of mRNAs. This observation resembles that reported for mature bull sperm, where the number of differentially expressed lncRNAs is also much greater than that of mRNAs^49^. It will therefore be interesting to investigate in the future whether such large number of lncRNAs in the seabream mature spermatozoon reflects different roles of these molecules in motility and early embryo development as suggested for mammals.

The transcriptomic analysis carried out here identified many DEGs between HGC and SPZ_EJ_, indicating that substantial changes in gene expression occurred during the transition between these two germ cell stages. The analysis however also revealed a higher heterogenicity in gene expression in SPZ_EJ_ than in HGC, suggesting that sperm cells at different stage of maturation may be present in the first population, thereby resulting in different cohorts of motile spermatozoa. In fact, the motility and progressivity of SPZ_EJ_ from different males at 5 s postactivation ranged from 88 to 98% and from 16 to 51%, respectively, while the time during which spermatozoa remained motile ranged from 5 to 8 min, which could be the consequence of differences in gene expression. These observations thus raise the possibility that specific transcripts are directly related to sperm motility in teleosts, and may be used as potential targets in fertility biomarkers as in mammals^41^.

In SPZ_EJ_, different genes involved in the cytoskeleton and ion and water transport were upregulated, which can be expected since sperm cells need to differentiate the flagellum and an intracellular signaling machinery in order to activate and maintain motility in the aquatic environment. The upregulated cystokeletal genes were mostly related to actin remodeling, such as *marcks* and other actin binding genes, and components of the kinesin motor complex, such as *kif20a* and *sybu*, which may be involved in the maturation of spermatozoa^50^. However, many components of the axoneme, which are likely necessary for flagellar function in piscine spermatozoa as in mammals^51–54^, such as a number of cilia- and flagella-associated proteins (*cfap43, -57, -61, -70*), sperm flagellar proteins (*spef1* and *-2*) and different dynein motor proteins, except dynein heavy chain 12 (*dnah12*) and cytoplasmic dynein 1 intermediate chain 2 (*dync1i2*), were downregulated in SPZ_EJ_. This observation suggests that these transcripts might be transiently upregulated during the differentiation and/or maturation of spermatozoa in the extratesticular ducts (ETDs) prior to ejaculation, although this needs further confirmation. Regarding membrane channels, *aqp7* was the most highly upregulated in SPZ_EJ_, which also accumulated transcripts encoding *aqp3a* and *-11*, suggesting that water and/or uncharged solute transport may be important during sperm formation. The ion channels and exchangers upregulated include the transient receptor potential cation channel subfamily V member 1 (*trpv1*), different potassium voltage-gated ion channels (*kcnc4*, *kcng4*, *kcnh1*, *kcnk6*) and the sodium potassium calcium exchanger 2 (*slc24a2*), which may be involved in the activation and maintenance of sperm motility^55,56^ or in the late stages of spermatogenesis^57^. Other ion channels potentially implicated in cell volume regulation upon activation of sperm in the hyperosmotic seawater, such as voltage-dependent anion-selective channel protein 1 (*vdac1*), volume-regulated anion channel subunit Lrrc8d (*lrrc8d*) and the mechanosensitive ion channel Piezo 1 (*piezo1*)^58,59^, were also highly accumulated in SPZ_EJ_.

An important process during the differentiation of spermatozoa is to prepare the cells to become fertilization competent, and therefore genes involved in sperm-egg interaction are likely to be activated. Accordingly, different genes encoding for cell adhesion proteins potentially involved in this mechanism were upregulated in SPZ_EJ_, such as several C-type lectins and cell surface glycoproteins (*cd209c*, *cd209e*, *cd200r1a*) and immunoglobulin receptors (*pigr*, *fcgr2a* and *-3*, *ildr1*). Interestingly, we also found two transcripts with sequence homology to zona pellucida (ZP) proteins highly upregulated in SPZ_EJ_ (log2 fold change > 9), one that seems to be the ortholog of mammalian ZP3 (*zp3*; XM_030443218.1) and another distantly related to ZP2 (*zp2*; AY928799.1). The expression of ZP proteins in mammals and fish has been originally thought to be ovary specific, where they are synthesized by the liver under estrogenic regulation and/or in the oocyte, and further deposited in the vitelline envelope separating the oocyte from the somatic cells which will form the chorion^60,61^. Recent studies, however, have found the expression of ZP3 proteins in spermatogonia, spermatocytes and round and elongated spermatids, but not in spermatozoa, in both human and mouse^62^. Therefore, our findings are not completely surprising, but they may reflect the presence of remnant mRNAs from the processes of spermatozoa differentiation rather than mRNAs playing a role during sperm-egg recognition. In any event, further studies are necessary to confirm the presence of ZP proteins in fish spermatozoa and elucidate their potential function.

An intriguing observation of the present study was the high number of upregulated transcripts related to transcription and translation found in SPZ_EJ_. Many of these mRNAs encode for transcription factors, transcriptional and translational regulators, and ribosomal proteins, including seven different mitochondrial ribosomes. Vertebrate spermatozoa are believed to be trancriptionally and translationally silent as a result of the degradation of ribosomal RNAs and the gradual replacement of the histones of the DNA-packing nucleosomes by protamines during the spermiogenic differentiation phase^63,64^. The many types of RNAs still present in the sperm are thus thought to be the remnants of the high transcriptional activity of the spermatocytic and spermatidogenic phases^63^, or delivered via epididymal extracellular microvesicles, called exosomes or epididysomes^65^. However, some studies suggest that mitochondrial ribosomal pathways, rather than the canonical cytoplasmic mechanisms, remain active in the spermatozoon and yield paternal factors that are important for sperm maturation, capacitation in the female reproductive tract, fertility, and early zygotic development^66–69^. As in several lineages of anamniotes^70–72^, the seabream spermatozoa are devoid of protamines^34^, which agrees with the failure to detect protamine-encoding mRNAs in our transcriptome. This observation, together with the high accumulation of transcription and translation related genes in seabream SPZ_EJ_, raises the question of whether transcriptional quiescence is a general feature of post-meiotic sperm maturation in teleosts. However, fish also produce exosomes in the ETDs as mammals^73^, and therefore it is also plausible that these spermatozoon mRNAs, as well as the lncRNAs, are originated from exosomes released from cells lining the ETDs for further function in early embryogenesis. Future studies should investigate the chromatin architecture reorganization and epigenetic marks in seabream spermatozoa that might allow transcription and translation during the maturation phase in the ETDs.

Previous studies in teleosts have shown that the energy-supplying pathways in spermatozoa include glycolysis, phospholipid catabolism and triglyceride metabolism, the TCA cycle, and OXPHOS, the latter two mechanisms being the key metabolic pathways that sustain basal metabolism^52,74^. Studies in the seabream suggest that lipids are the major substrate for ATP/energy production via the mitochondrial TCA cycle, while cytosolic glycolysis and carbohydrate metabolism seems to have a lesser contribution^75^. In contrast, upon motility activation OXPHOS seems to be the main ATP source for flagellar movement, which achieves a balance between energy production and consumption^76,77^. According to this view our interactome analysis showed that enzymes involved in gluconeogenesis were downregulated in SPZ_EJ_, while those playing distinct roles in the TCA cycle and OXPHOS, as well as in triglyceride synthesis and lipid catabolism, were in general upregulated. However, we also found that many glycolytic enzymes were upregulated in SPZ_EJ_, including lactate dehydrogenase (*ldha* and *ldhb*) involved in the conversion of pyruvate to lactate. This observation thus suggests that glycolysis may be an important metabolic pathway during the maturation of spermatozoa in the ETDs since this pathway is more effective under anaerobic than aerobic conditions.

Finally, the in silico analysis of DEGs in SPZ_EJ_ revealed the enrichment of different signaling pathways, amongst which the cytokine/chemokine, GnRHR and PDGF pathways appear the most predominant. Evidence for the existence of chemotaxis in fish, guiding the spermatozoa to the vicinity of the egg and even orienting the direction of their swimming path toward the egg micropyle, is increasing^74,78–81^, and therefore our observation of the presence of almost all components of the cytokine/chemokine signaling pathway in SPZ_EJ_ is consistent with these findings. The expression of the GnRHR and PDGF pathways in spermatozoa, including mRNAs encoding Gnrh and Pdgf cognate receptors, is however more compelling. Previous studies in teleosts have shown that administration of some hormones, such as progestins, androgens and gonadotropins, can increase the seminal plasma pH in the ETDs, which results in the elevation of intra-sperm cAMP levels, increase hydration, or induce the secretion of sperm-immobilizing ions by the ETD epithelium^2,82^. However, the cellular sources of these hormones in the ETDs and their potential signal transducing effects in the maturing spermatozoa are completely unknown. Nevertheless, it can be speculated that the expression of the GnRHR and PDGF pathways in seabream spermatozoa may reflect a prior function of these factors as paracrine signals in the ETDs for inducing the maturation and acquisition of full motility of spermatozoa, which might involve the activation of transcription and/or translation of specific genes as discussed above. This challenging hypothesis is not yet proven and merits further investigation.

In summary, the transcriptome dynamics during spermiogenesis described for the first time here provide important insights into the molecular mechanisms underlying sperm differentiation and maturation in non-mammalian vertebrates. Our data uncovered a number of candidate genes, lncRNAs and novel endocrine pathways that may play important roles for the acquisition of the spermatozoon motility and during early embryo development of teleosts. Further studies will be necessary to dissect out the specific functions of these genes during the transition of haploid cells to spermatozoa, as well as during the subsequent maturation in the extratesticular tract.

## Methods

### Animals and sample collection

Adult gilthead seabream males were raised in captivity at the Institut de Recerca i Tecnologia Agroalimentàries (IRTA) aquaculture facilities in San Carlos de la Rápita (Tarragona, Spain) and maintained in the laboratory as described previously^38^. Samples of testis and SPZ_EJ_ were obtained from males during the natural reproductive season (November-February). The sperm was collected from males sedated with 500 ppm of phenoxyethanol (Merck) by the application of a soft pressure to the abdominal area and removal of sperm from the gonopore with a syringe, while the testes were collected from anaesthezized fish and euthanized by decapitation. Procedures relating to the care and use of animals and sample collection were approved by the Ethics Committee (EC) of Institut de Recerca i Tecnologia Agroalimentàries (IRTA), following the International Guiding Principles for Research Involving Animals (EU 2010/63), and in accordance with ARRIVE guidelines (https://arriveguidelines.org).

### Cell cytometry

To isolate HGC by FACS, testicular biopsies were cut into small pieces of ~ 1 g and treated with 0.2% collagenase (Merck type 1A) for 1 h under agitation in non-activating medium (NAM; in mg/ml: 3.5 NaCl, 0.11 KCl, 1.23 MgCl_2_, 0.39 CaCl_2_, 1.68 NaHCO_3_, 0.08 glucose, 1 bovine serum albumine [BSA], pH 7.7; 280 mOsm) (*51*) supplemented with 200 μg/mL penicillin/streptomycin (Life Technologies Corp.). Samples were centrifuged at 200× *g* for 1 min to remove cell aggregates, and the supernatant centrifuged again at 400× *g* for 1 min to enrich in haploid cells. The cells were centrifuged at 400× *g* for 5 min and the pellet resuspended in 1 ml NAM. The concentration of cells was determined by light microscopy and the ISASv1 software (Proiser), and this was adjusted to 150 × 10^6^ cells/ml. Cells were then stained with 200 nM of a solution of SYBR Green I (SGI) fluorescent nucleic acid stain (Molecular Probes, Life Technologies Corp.) for 45–60 min in the dark at room temperature, just prior to flow cytometry.

FACS was performed with a MoFlo XDP cell sorter (Beckman Coulter) equipped with three lasers (blue solid state of 488 nm, red diode of 635 nm, and argon ion UV laser of 351 nm). Sterilized PBS served as the sheath fluid. The sorter was set in 4-way purify sort mode and with a flow sorting rate of ~ 1500 events/s. The sorted population of HGC was collected in 4 ml of NAM in 15 ml tubes and centrifuged at 200× *g* for 15 min. The resulting pellet was resuspended in 100 μl of NAM to obtain aliquots of 3 to 5 × 10^6^ cells, which were centrifuged again at 200× *g* and frozen in liquid nitrogen and stored at − 80 °C.

### Immunofluorescence microscopy

Sorted germ cells and SPZ_EJ_ were processed as described previously^7,38^ and attached to UltraStick/UltraFrost Adhesion slides (Electron Microscopy Sciences). Samples were fixed in 4% PFA in PBS for 15 min before antigen retrieval in three consecutive 5-min incubations with boiling citrate (10 mM at pH 6), followed by triton X-100 (0.2% in PBS) for 15 min. After blocking for one hour in PBST with 5% normal goat serum (Merck G9023) and 0.1% BSA, antibodies were applied overnight at 4 °C in a humidified chamber. The primary antibodies were α-tubulin (Merck T9026; 1:1000), H3K9ac (Abcam ab4441; 1:1000), and Spo11 (Santa Cruz Biotechnology sc-33146; 1:1000). Anti-mouse or anti-rabbit IgG coupled with Alexa-555 (Invitrogen A-21422 and Merck AP510C, respectively) were applied for 1 h at room temperature and cells were counterstained with 4′,6-diamidino-2-phenylindole dihydrochloride (DAPI; Merck G8294; 1:3000) before mounting with Fluoromount™.

### Evaluation of sperm motility by CASA

The percentage of motile and progressive spermatozoa, as well as the time during which the sperm remained motile, was determined by CASA using the Integrated Semen Analysis System (ISASv1, Proiser) software as previously described^83^.

### RNA extraction, library construction, and sequencing

Total RNA from HGC (3 × 10^7^ cells) and SPZ_EJ_ (3–30 × 10^7^ cells) was extracted with the RNeasy Plus Mini Kit (Qiagen). The full-spectrum UV–Vis spectro-photometer NanoDropVC 2000 (Thermo Fisher Scientific) was used to determine the purity and concentration of the extracted RNA by measuring their 260/280 nm absorbance ratio. RNA size distribution profiles were analyzed using the Agilent 2100 Bioanalyzer (Agilent Technologies). The RIN values ranged from 8.4 to 7.5 in HGC, whereas these values ranged from 1 to 3.2 in SPZ_EJ_. The absence of peaks corresponding to 28S and 18S rRNAs in SPZ_EJ_ was confirmed to verify the absence of non-sperm cells in these samples.

Four unstranded RNA libraries (replicates) for low-input RNA were constructed for each of the HGC and SPZ_EJ_ groups; each replicate being a pool of cells collected from three different males. The libraries from the total RNA were prepared following the SMARTseq2 protocol for low-input RNA^84^ with some modifications. Briefly, reverse transcription with 2 ng RNA was performed using SuperScript II (Invitrogen) in the presence of oligo-dT30VN (1 µM; 5′-AAGCAGTGGTATCAACGCAGAGTACT_30_VN-3′), template-switching oligonucleotides (1 µM) and betaine (1 M). The cDNA was amplified using the KAPA Hifi Hotstart ReadyMix (Merck), 100 nM ISPCR primer (5′-AAGCAGTGGTATCAACGCAGAGT-3′) and 12 cycles of amplification. Following purification with Agencourt Ampure XP beads (1:1 ratio; Beckmann Coulter), product size distribution and quantity were assessed on a Bioanalyzer High Sensitvity DNA Kit (Agilent). The amplified cDNA (200 ng) was fragmented for 10 min at 55 °C using Nextera® XT (Illumina) and amplified for 12 cycles with indexed Nextera® PCR primers. The library was purified twice with Agencourt Ampure XP beads (0.8:1 ratio) and quantified on a Bioanalyzer using a High Sensitvity DNA Kit.

The libraries were sequenced on HiSeq2500 (Illumina) in paired-end mode with a read length of 2 × 76 bp using TruSeq SBS Kit v4. We generated more than 30 million paired-end reads for each sample in a fraction of a sequencing v4 flow cell lane, following the manufacturer’s protocol. Image analysis, base calling and quality scoring of the run were processed using the manufacturer’s software Real Time Analysis (RTA 1.18.66.3) and followed by generation of FASTQ sequence files by CASAVA 1.8.

### Genome annotation

To improve the gilthead seabream reference genome^37^ annotation for the differential expression analysis, the genome was reannotated, and a de novo transcriptome assembly was generated from which those transcripts not present in the genome assembly were added to the analysis.

#### Genome reannotation

Repeats present in the seabream genome assembly were annotated with RepeatMasker v4-0-7 (http://www.repeatmasker.org) using the zebrafish repeat library included in RepeatMasker. The gene annotation was obtained by combining transcript alignments, protein alignments and ab initio gene predictions. First, the RNA-seq reads were aligned to the genome with STAR v-2.5.3a^85^. Subsequently, transcript models were generated using Stringtie v1.0.4^86^ and PASA assemblies were produced with PASA v2.0.2^87^ by adding also the 114,155 *S. aurata* ESTs present in NCBI (October 2017). Secondly, the complete Actinopterygii proteomes were downloaded from Uniprot in October 2017 and aligned to the genome using Spaln v2.4.7^88^. Ab initio gene predictions were performed on the repeat masked assembly with three different programs: GeneID v1.4^89^, Augustus v3.2.3^90^ and Genemark-ES v2.3e^91^ with and without incorporating evidence from the RNA-seq data. The gene predictors were run with trained parameters for human except Genemark that runs on a self-trained manner. Finally, all the data was combined into consensus CDS models using EvidenceModeler-1.1.1^87^. Additionally, UTRs and alternative splicing forms were annotated through two rounds of PASA annotation updates. Functional annotation was performed on the annotated proteins with Blast2go^92^, using Blastp^93^ search against the nr database (March 2018) and Interproscan^94^ to detect protein domains on the annotated proteins.

The annotation of non-coding RNAs was carried out using the following steps. First, the program cmsearch v1.1^95^ included in the Infernal software^96^ was run against the RFAM v12.0 database of RNA families^96^. The tRNAscan-SE v1.23^97^ was also run to detect the transfer RNA genes present in the genome assembly. To detect the lncRNAs we selected those PASA-assemblies that had not been included into the annotation of protein-coding genes in order to get all those expressed genes that were not translated into a protein. Finally, those PASA-assemblies without protein-coding gene annotation that were longer than 200 bp and whose length was not covered at least in an 80% by a small ncRNA were incorporated into the ncRNA annotation as lncRNAs. The resulting transcripts were clustered into genes using shared splice sites or significant sequence overlap as criteria for designation as the same gene.

#### *Complementing the annotation with *de novo* assembled transcripts*

The RNA-seq reads were assembled with Trinity v2.2.0^98^ allowing for trimming and normalization of the reads. Next, Rapclust v0.1^99^ was run, in which the process of pseudoalignment was first performed with Sailfish v0.10.0^100^, and then Rapclust was used to cluster the assembled sequences into contained isoforms in order to reduce redundancy and to cluster together all the isoforms that are likely to belong to the same gene. For evaluation of the resulting transcriptomes we estimated their completeness with BUSCO v3.0.2^101^ using an Actinopterygii specific dataset of 4584 genes. After obtaining the reference transcriptome, open reading frames (ORFs) were annotated in the assembled transcripts with Transdecoder^102^ and functional annotation was performed on the annotated proteins with Blast2GO, as described above. Finally, the assembled transcripts were mapped against the seabream reference genome assembly with GMAP^103^. Those transcripts for which less than 50% of their length aligned to the genome, and with a complete ORF and functional annotation, were added to the reference genome as separate annotated contigs.

### Differential expression analysis

RNA-seq reads were mapped against the improved version of the seabream reference genome with STAR v2.5.3a using ENCODE parameters for long RNAs. Genes were quantified with RSEM v1.3.0^104^ using the improved annotation. Sample similarities were inspected with a PCA using the top 500 most variable genes and the 'rlog' transformation of the counts. Differential expression analysis was performed with DESeq2 v1.18^105^ with default options, and genes with a false discovery rate (FDR) < 1% were considered significant. Heatmaps with the ‘rlog’ transformed counts of the DEGs were plotted with the ‘pheatmap’ R v1.0.12 package available at the Rstudio v1.2.1335 (http://www.rstudio.com/). Venn diagrams and volcano plots were performed with the ‘VenDiagramm’ v1.6.20 R package and ‘ggplot2’ v3.1.1 R package, respectively, from Rstudio v1.2.1335.

### Gene classification, ontology, and pathway analysis of DEGs

The GO enrichment of DEGs and signaling pathway analyses were performed using the PANTHER v14.1 Classification System and analysis tools (http://www.pantherdb.org/). GO terms and pathways with FDR < 0.05% were considered significant. Scatter plots of pathway analyses were carried out with ‘ggplot2’ R package. Functional category classifications were also done manually using the Uniprot database (https://www.uniprot.org/) and QuickGO browser (http://www.ebi.ac.uk/QuickGO). Interactome analyses were conducted using the STRING database v11.0b^39^ with a high-confidence interaction score (0.9), and plots were performed using Cytoscape v3.8.2 (https://cytoscape.org/). In some cases, selected transcripts were mapped using the KEGG pathway database^106^ (https://www.genome.jp/kegg/pathway.html) and WikiPathways (https://www.wikipathways.org).

### Validation of gene expression by qRT-PCR

The qRT-PCR were carried out as described previously^7,38^, except that in this case the cDNA was synthesized from 13 to 20 ng of total RNA using the AccuScript High-Fidelity 1st Strand cDNA Synthesis Kit (Agilent 200,820) following the manufacturer’s instructions. For qRT-PCR, relative gene expression levels with respect to HGC were determined by the 2^−ΔΔCt^ method, using glutathione-specific gamma-glutamylcyclotransferase 1 (*chac1*) and beta-actin (*bactin*) as reference genes. The analyses were done on three cDNAs synthesized from three different pools of three animals each using technical duplicates. Primer3 v. 0.4.0 software (https://bioinfo.ut.ee/primer3-0.4.0/) was used for primer design. Primer sequences are listed in Suppl. Table S6.

## Supplementary Information

Supplementary Information 1.

Supplementary Information 2.

Supplementary Information 3.

## Data Availability

The RNA-seq datasets generated in this study have been submitted to Gene Expression Omnibus (GEO) database at the National Center for Biotechnology Information (NCBI) under accession no. GSE173088. Reannotation data from the seabream genome are available at https://denovo.cnag.cat/Saurata.

## Abbreviations

CASA: Computer-assisted sperm analysis
DEGs: Differentially expressed genes
ETDs: Extratesticular ducts
FACS: Fluorescence-activated cell sorting
GnRHR: Gonadotropin releasing hormone receptor
GO: Gene ontology
HGCs: Haploid germ cells
lncRNA: Long non-coding RNAs
OXPHOS: Mitochondrial oxidative phosphorylation
PCA: Principal component analysis
PDGF: Platelet-derived growth factor
qRT-PCR: Real-time quantitative reverse transcription PCR
RIN: RNA integrity number
SPZ_EJ_: Ejaculated spermatozoa
SPZ_I_: Intratesticular spermatozoa
TCA: Tricarboxylic acid cycle
ZP: Zona pellucida
