# Supplementary material for: Developmental RNA-Seq transcriptomics of haploid germ cells and spermatozoa uncovers novel pathways associated with teleost spermiogenesis
Source: Sci Rep. 2022 Aug 19;12:14162. doi: 10.1038/s41598-022-18422-2 (PMC9391476; doi:10.1038/s41598-022-18422-2)
Supplement: Supplementary file 1 — Supplementary Information 1. [file 41598_2022_18422_MOESM1_ESM.pdf]

Supplementary Information

Developmental RNA-Seq transcriptomics of haploid germ cells and spermatozoa uncovers novel pathways associated with teleost spermiogenesis

Júlia Castro-Arnau[1], François Chauvigné[1,2], Jessica Gómez-Garrido[3], Anna Esteve-Codina[3], Marc Dabad[3], Tyler Alioto[3,4], Roderick Nigel Finn[1,5] and Joan Cerdà[1]*

[1]Institute of Agrifood Research and Technology (IRTA)-Institute of Biotechnology and Biomedicine (IBB), Universitat Autònoma de Barcelona, 08193 Barcelona, Spain; [2]Institute of Marine Sciences, Consejo Superior de Investigaciones Científicas, 08003 Barcelona, Spain; [3]Centre Nacional d'Anàlisi Genòmica-Centre for Genomic Regulation, Barcelona Institute of Science and Technology, 08028 Barcelona, Spain; [4]Universitat Pompeu Fabra, 08003 Barcelona, Spain; [5]Department of Biological Sciences, Bergen High Technology Centre, University of Bergen, 5020 Bergen, Norway

Correspondence and requests for materials should be addressed to J.C. (joan.cerda@irta.cat)

Supplementary Figures

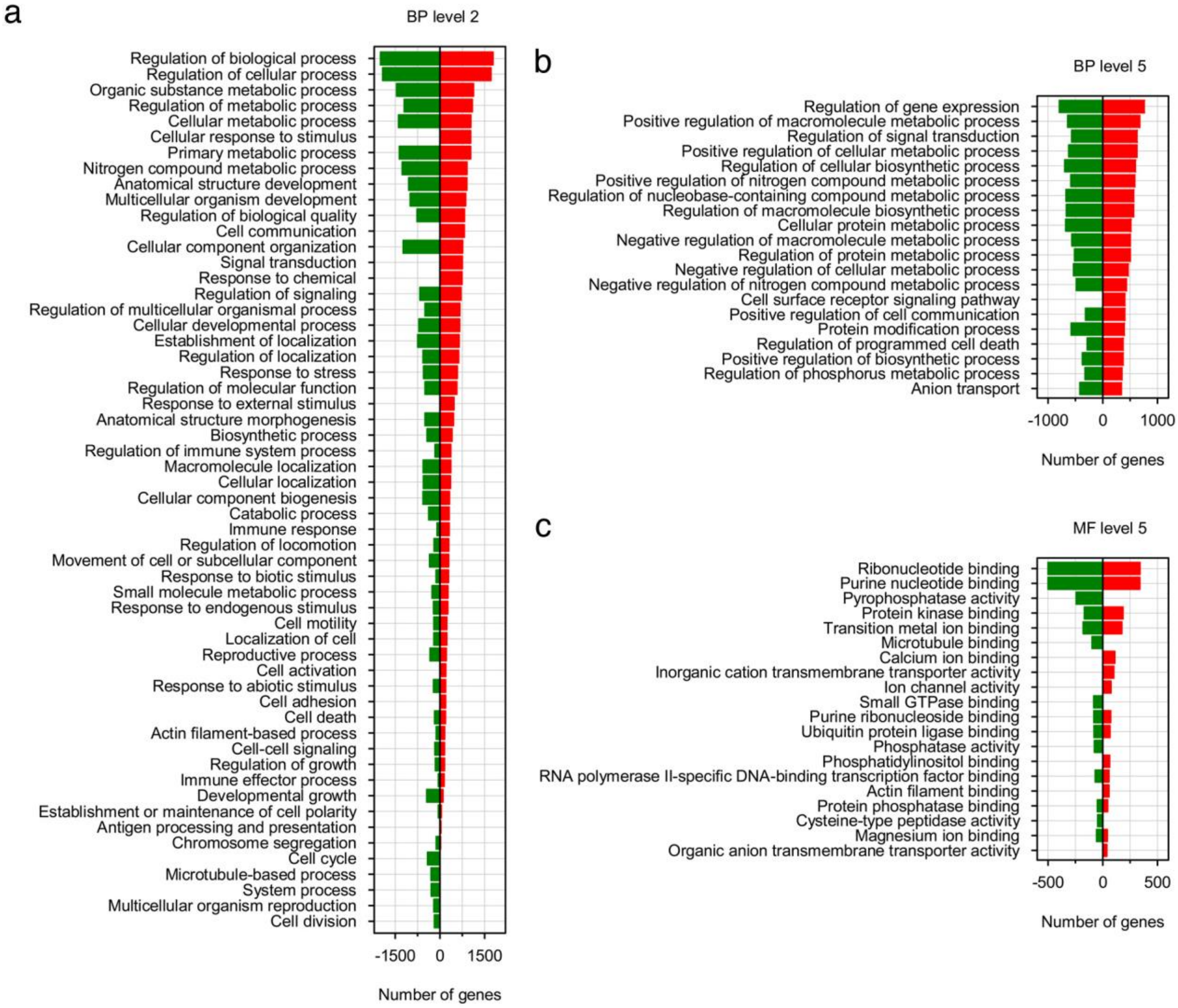

Figure S1. Gene ontology (GO) enrichment analysis of the DEGs during sperm differentiation and maturation. GO annotation of DEGs corresponding to biological process level 2 (**a**) and 5 (**b**), and molecular function level 5 (**C**). The horizontal axis displays the number of significant genes corresponding to each functional type, whereas the vertical axis displays the second level of GO annotation.

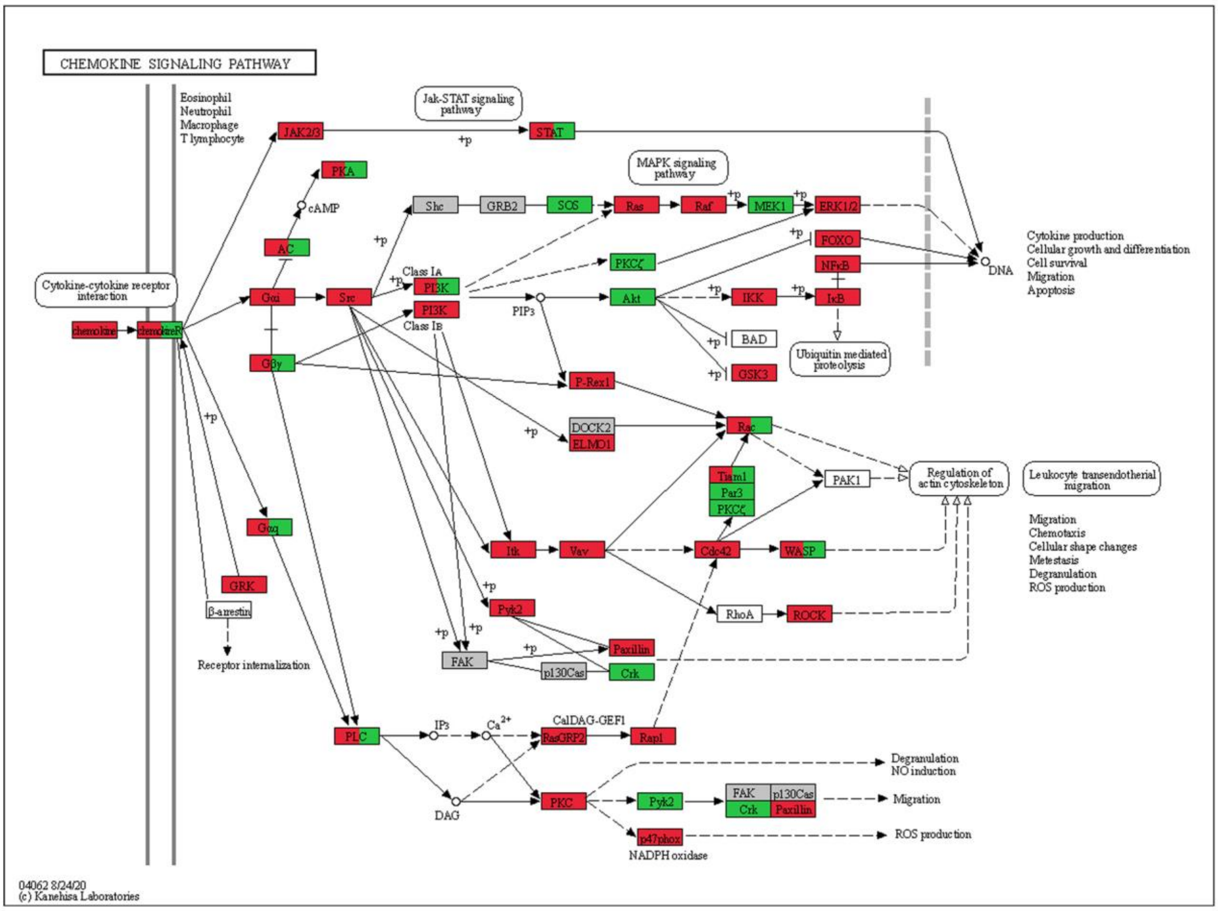

Figure S2. Mapping of DEGs and not regulated transcripts in the chemokine signaling pathway. Schematic diagram of the chemokine signaling pathway from the KEGG pathway database (https://www.genome.jp/dbget-bin/www_bget?pathway:map04062) showing the upregulated transcripts in red, downregulated transcripts in green, and not regulated transcripts in grey. Permission has been obtained from Kanehisa laboratories for using KEGG pathway database.

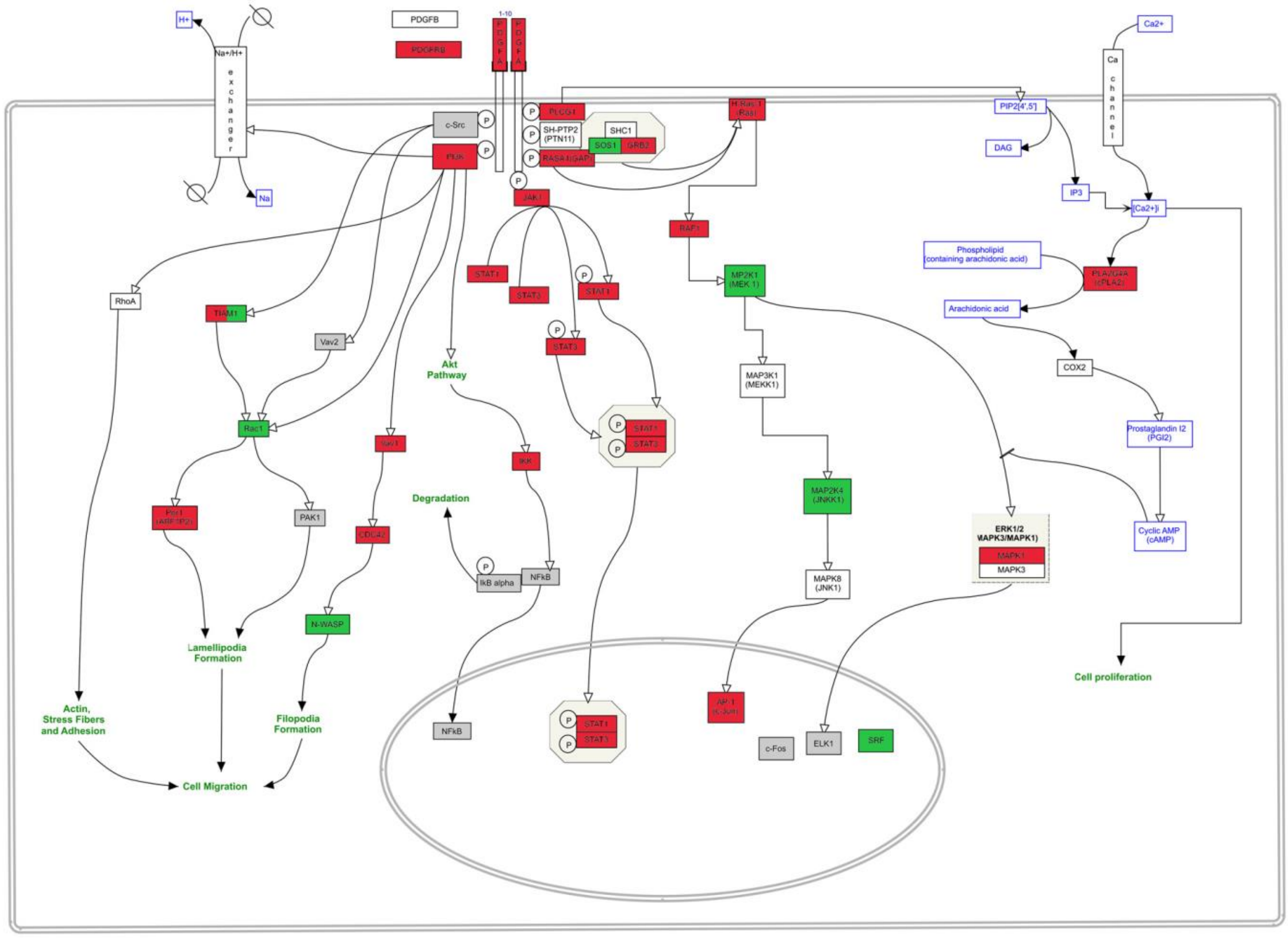

Figure S3. Mapping of DEGs and not regulated transcripts in the PDGF signaling pathway. Schematic diagram of the PDGF pathway from the WikiPathways database (https://www.wikipathways.org/instance/WP2526_r121697) showing the upregulated transcripts in red, downregulated transcripts in green, and not regulated transcripts in grey.

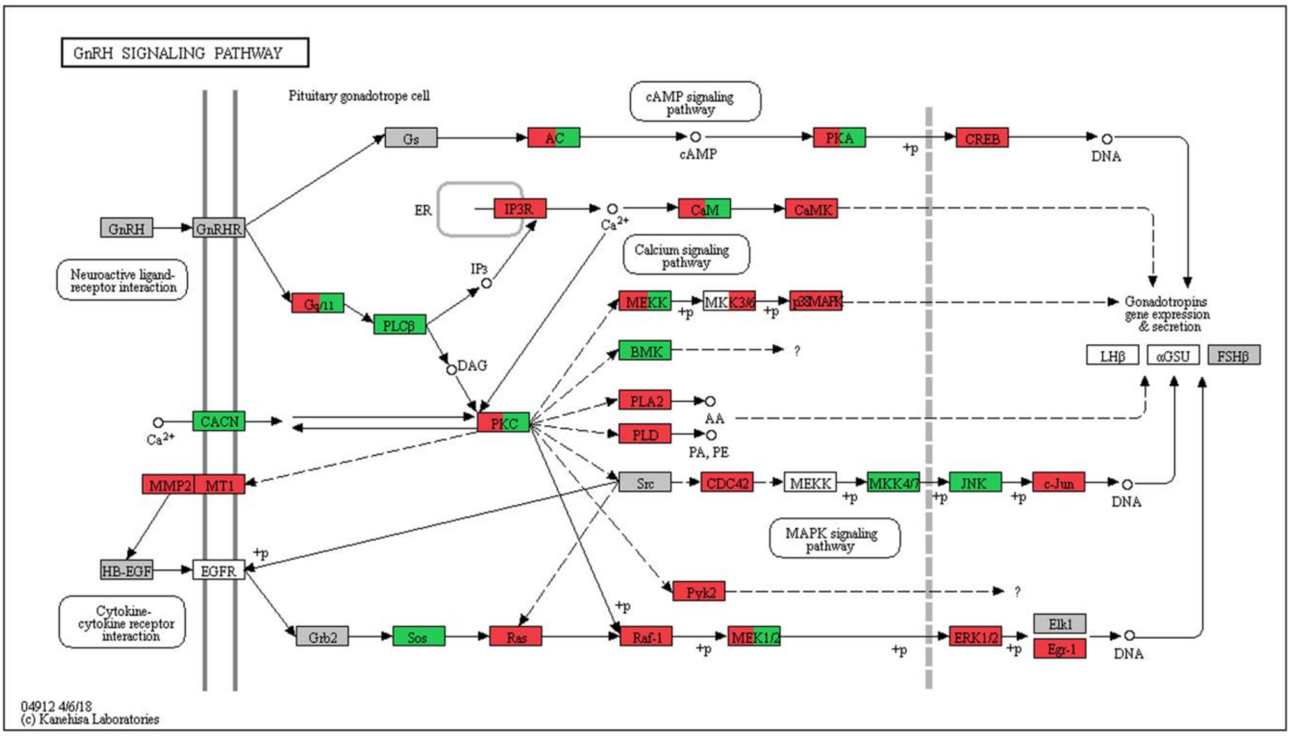

Figure S4. Mapping of DEGs and not regulated transcripts in the GnRH signaling pathway. Schematic diagram of the GnRH signaling pathway from the KEGG pathway database (https://www.genome.jp/dbget-bin/www_bget?pathway:map04912) showing the upregulated transcripts in red, downregulated transcripts in green, and not regulated transcripts in grey. Permission has been obtained from Kanehisa laboratories for using KEGG pathway database.

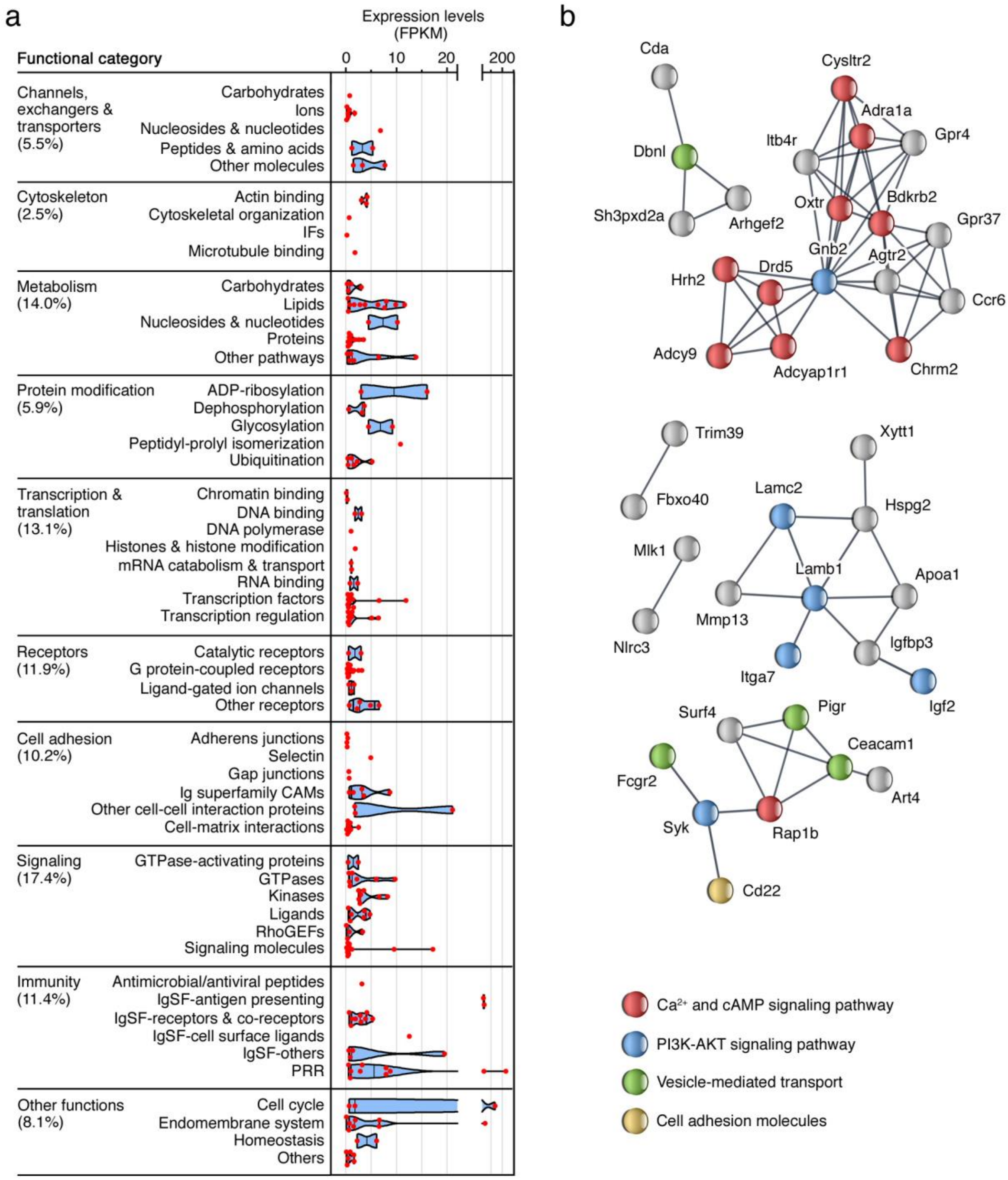

Figure S5. *In silico* functional analysis of transcripts emerging *de novo* in SPZ$_{EJ}$. (**a**) Plot showing the expression levels assessed as fragments per kilo base per million mapped reads (FPKM) of the 239 *de novo* transcripts classified into ten functional categories. The percentage of mRNAs in each category is indicated. PRR, pattern recognition receptors. (**b**) Interactions of proteins (network nodes) encoded by *de novo* mRNAs involved in Ca^{2+}/cAMP and PI3K-AKT signaling, vesicle-mediated transport, cell adhesion and other functions. The edges represent protein-protein associations predicted with the STRING v11.0b software with high confidence score (0.9). Interaction plots were generated with Cytoscape v3.8.2 (https://cytoscape.org/).

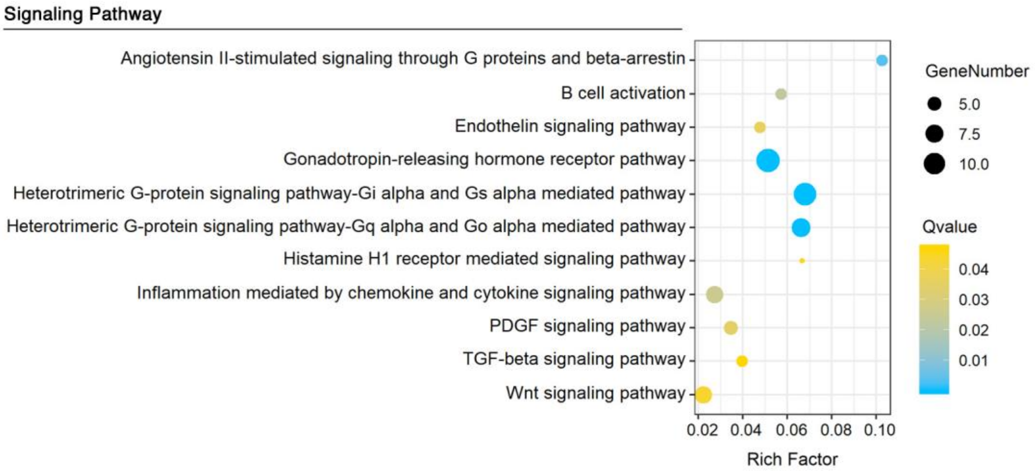

Figure S6. Gene set enrichment analysis among the genes accumulated *de novo* in SPZ_{EJ}. Pathway analysis was carried out using the PANTHER Classification System. The 11 most highly enriched signaling pathways (FDR < 0.05) are shown. The plot was generated with the 'ggplot2' R package (https://ggplot2.tidyverse.org).
