# Supplementary material for: Developmental RNA-Seq transcriptomics of haploid germ cells and spermatozoa uncovers novel pathways associated with teleost spermiogenesis
Source: Sci Rep. 2022 Aug 19;12:14162. doi: 10.1038/s41598-022-18422-2 (PMC9391476; doi:10.1038/s41598-022-18422-2)
Supplement: Supplementary file 3 — Supplementary Information 3. [file 41598_2022_18422_MOESM3_ESM.pdf]

Supplementary Table S6

Nucleotide sequences of the primers employed for qRT-PCR

GenBank accession	Transcript symbol	Forward sequence	Reverse sequence
XM_030407493	*aco2*	CCCCGATGACAAGATTTCAA	CAGCCTGGAACCACTCAATC
XM_030406939	*actb*	CCAAAGCCAACAGGGAGAAG	AGATGGGTACTGTGTGGGTCA
XP_030261683	*ak7*	GTTGGACTGCAGCAAGATCA	CTCATCACAAACGGAGCTGA
XM_030422127	*akt3*	GGAGACGGACACACGCTACT	GGAGAACTGTGGGAAATGAGG
XP_030292248	*alad*	CAGGGAATGTGTCCTGTGTG	GAGGCAGCAGTCACACGATA
XP_030255569	*aldoa-l*	CGGTGGAGAGTCCCTGTTT	GTTAGGCGTTAGTGCCGAAGT
XP_030286254	*alox5*	CCCAGGAGCAGATTGACAAG	TGAATCCCTCTTGGAACAGAC
KC589386	*aqp7*	TGAAGGACTTGGTGCAGTCA	CCTAGGCCAAACACCATCAT
XP_030249041	*ash1l*	AAGAGGAGAGAGGGCCAGAG	GTCACCCTCGGGTTTATCCT
XM_030439663	*braf*	GCTGTTGGCTCGCTCATTAC	GATAGGGGTTTTGGGTGAGG
XM_030404141	*casp8*	GGGGTGACGACATAACAACC	GAGCTTCATTTCTGTTGTGGTG
XP_030299278	*ccr6*	TCCGAGATCTGCGTGTCTACT	TTCCTGGTCCAGACAGATCC
XP_030299721	*ccr6-l*	GCTGTCGGGCAGAAAAGAT	CTCCGGAACTTTACCCCAAT
XP_030281295	*cdk10*	ACCCTGTGAACCAGAGCTGA	CCAGCTGTCTCATCATCACTG
XP_030261875	*chac1*	CGTGGATGACAATCACCTGT	GTGGAAAGCCTCTGTCGTGT
XP_030262161	*csc1*	GCCCAAACTTCAGCAGTCTTA	GATTTACGATCGGGAGTTGC
XP_030250969	*ctss*	ACCACGCTGTGTTAGCTGTG	CAGAGTGATGCCACACTGGT
XM_030395723	*cxcr5*	CCACTTTCGCAATGAGCTG	TGGAGCTGGTGGTTGTTACTC
XP_030293664	*egr1*	CCCCACCTCTACTCCTCCTC	AGGTCAGAGGTCACGACTGG
XM_030411705	*elmo1*	CTCCTGGGGAAGGAGATGAC	CTGGGTTCTTTGGGAATCG
XM_030436654	*fbp1*	CGATGACGTGCAGGAGTATCT	TGCAGCTGAAGGAGTGAGTG
XM_030399511	*g6pc3*	CGTCCCTGGACTGGTTCAT	CCCACAAGAGTACCCGTGAA
XP_030279426	*g6pd*	GGTGGCCAGAGGGAAATAGT	ATAGAGCTCCCTCCTGAGCTG
XP_030259080	*glul*	GCCAGGAGAAGAAGGGCTAC	TAAAGTCAGGAGGGCAGTGG
XP_030271495	*gnrh1r*	CCAAACGACTGAGAAAGGACA	AACGGTGTTGTTGTCGAGGT
XP_030269490	*gnrh2r*	CTGCTTGGATCCCATCATCT	GGAGAACCACATCTGCCTGT
XP_030280787	*gnrh3r*	CCGTAAGTCGAGAGATGTGC	CCTTCTGTTTCACTCCTCGTG
XP_030281022	*gpi-l*	CTGTCTGTGTGGTCGTCAGG	CTGGTAGAAGGCGTGCTGTC
XP_030253049	*gpx3*	GTTCTGGCAGCCTATGAAGC	GAGCAGGTATCTGCGGATGT
XP_030274648	*itpr1*	AGCAGAGGAAGCAGAAGCAG	GCACAGTCAGGAGTGCTCAG
XP_030281751	*itpr2*	AGCAGATGACGGAACAGAGG	GGCTTGAGTTTGTGTCGTCTT
XP_030290448	*jak1*	GTTGAGCAGGCAACTCTGTG	AGTGTAACAGCCGCTGAGGT
XM_030416057	*jak2*	TTTGCGTCCTTCTTTCAAGG	TTAAGAGCGACTCGGCTGAC
XP_030290270	*jun*	GAAGGTGATGAACCACGTCA	CGTTTCGTTCAACCACAGTC
XP_030278504	*kcnc4*	ACCCCAACGTCCATACTGAG	CAGCTCTGCACAGCGTCTAC
XP_030272802	*kin1ae*	CAGGACTCTCTGGGTGGAAA	TCTTTGCTGTCCACATTTCG
XP_030259646	*lrrc8d*	GTTCAGATGCACCAAGCTCA	TAAAGGGTGCGTTGCTCTCT
XP_030263907	*map2k4*	GACGAGTCAAAAAGGCCAAA	CATCGGTGAGCTGGGAGA
XP_030256967	*mapk7*	CTCTCGGCCTCCCTCTTATC	TCAGGGGGAATGGTATCAGA
XP_030281999	*mapk12*	GACCACTCGGTGGACAACTC	CTGCACAGTCGAGTCTTCGT
XP_030287334	*mmp14*	GAGCGCTTTTGTTCTTCAGG	CCTCGTTCTCTGTCCCTCTG
XP_030260647	*mrpl19*	TCCAAACTGGAGGAGCAGAT	GATCAGCAGCAGTGTTCAGC
XP_030251358	*pik3cb*	ACTCACGACTTCATCCACGTC	CATGAGGGCAAACAGTGTGA
XP_030294082	*pdgfrb*	AGCCCTCAGCTGTCAGAATC	GGTCTGAAAGGCAGCTGAAC
XP_030255317	*pdk1*	TCCTGAAAGGGGAGATTCCT	CCTGGATCTTCTTGCACCAT
XP_030271603	*pgam1*	GCGCTCTTTTGACACTCCTC	CTGGCGATGGTATCCTTCAG
XM_030441586	*pgam1-l*	GCTCGACAAGAACCTGAAGC	GAGGCGACTTCTCAGTCACTTT
XP_030271414	*pkm*	GATGTGTGGGCAGAGGATGT	GCGCATGGTGTTGGTGTA
XM_030424984	*plcz1*	CACTGGGATGCTGACATGAA	CAGCGGTAGCCTCTCTTCAG
XP_030292336	*prkaa1*	GGAAGCCACACCATTGAGTT	CGGTTGCATGCTTATTGCTA
XP_030268357	*prkcb*	CCCCCAGATCAGGAGCTTAT	TCTGAGGTGTGGGGGATTAC
XP_030287375	*ptgdr2*	CAGCACCAGTGAGACTGCAT	CCTGGGCACATTAACATACG
XP_030296439	*ptprr*	AGCCGACAGAGGAGGTATGA	GCACACTTCCTGGTTCAGGT
XP_030277815	*raf1*	GCGAATGAGGGAATCCTTAAC	AACACCCAGCCACTGAAGAT
XP_030254189	*rec8*	TGCAGCTCACACCCTCTACA	GCCTAAAGGACTAGCGTTGG
XP_030287257	*tesk1*	AAGAACTTGAATGGGGACCA	TTGGTGCAGAGTTTCGACAT
XP_030289820	*tie1*	GCCAGGAAGGCTTATGTGAA	GGACTTTCTCCTCGGGCTAC
XP_030256345	*timp2*	TGCTCCTGTGTCTGCTGTCT	CCTTCTTCTGGGTGTTGCTC
XM_030423559	*tkt2*	ACCTTTGCAAACTGAGGACTGT	ACAATAGGTGCACACAGTGGAG
XP_030252175	*tlr1*	CAACCAGCTGAAGTCCATGA	GGGTAGATGGATTTGCCTCA
XP_030265677	*tuba4a*	GGATGGAGGAGGGAGAGTTC	GGCTTTTCTTTGTGGCAGAT
XP_030251226	*spdl1*	CCCCAGCTCTCCTGTAAGTG	TGCTGAGCACATTGGTTTTC
XP_030281360	*slc28a2*	CCTCCACTTGACTGTGTGGA	TTCAGACCAGGAAGCTCACA
XP_030262590	*stat3*	CTGATGCACAATGAAGCTGAA	GGGAGAATCCTCCATCACAAC
XM_030414700	*vav1*	GCCAACTATGTGGAGGAGGA	GTGCACATGGGTTAATGCAA
XP_030276225	*vdrb*	GCTCTACGCCAAGATGATCC	GCAGCCTCTAGGAGACTTCG
